# Supplementary material for: Neuroanatomical Correlates of Negative Symptoms in Schizophrenia
Source: bioRxiv. 2025 Sep 23:2025.09.22.677864. Preprint. [Version 1] doi: 10.1101/2025.09.22.677864 (PMC12485849; doi:10.1101/2025.09.22.677864)
Supplement: Supplement 1 [file media-1.pdf]

Supplementary Figure 1a. Pattern of Mean Correlations Between Cortical Thickness and SANS Total, MAP and EXP

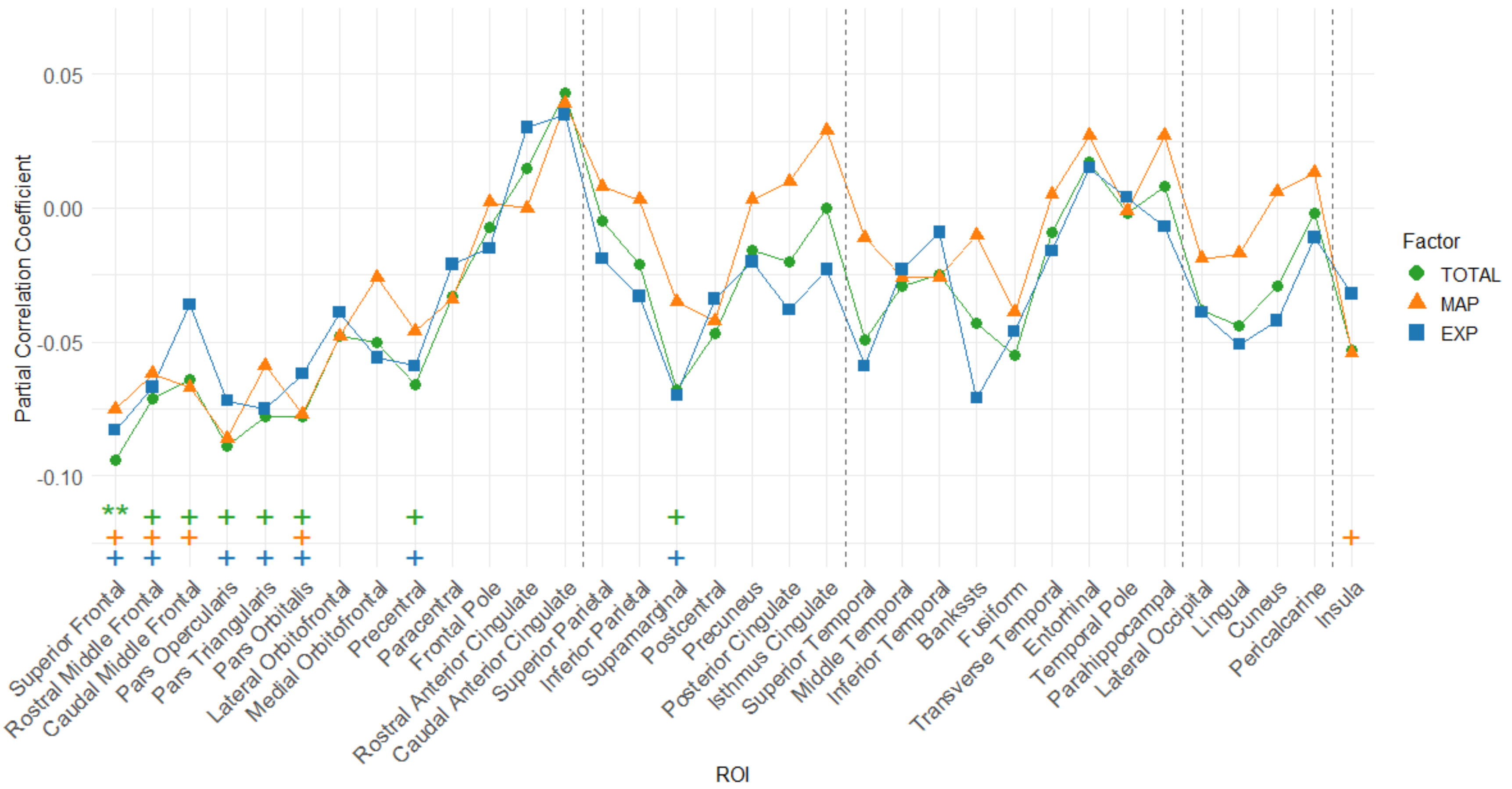

Note: FDR significant denoted with ** and nominal significance denoted with +

Supplementary Figure 1b. Pattern of Mean Correlations Between Cortical Thickness, and MAP dimensions and domains

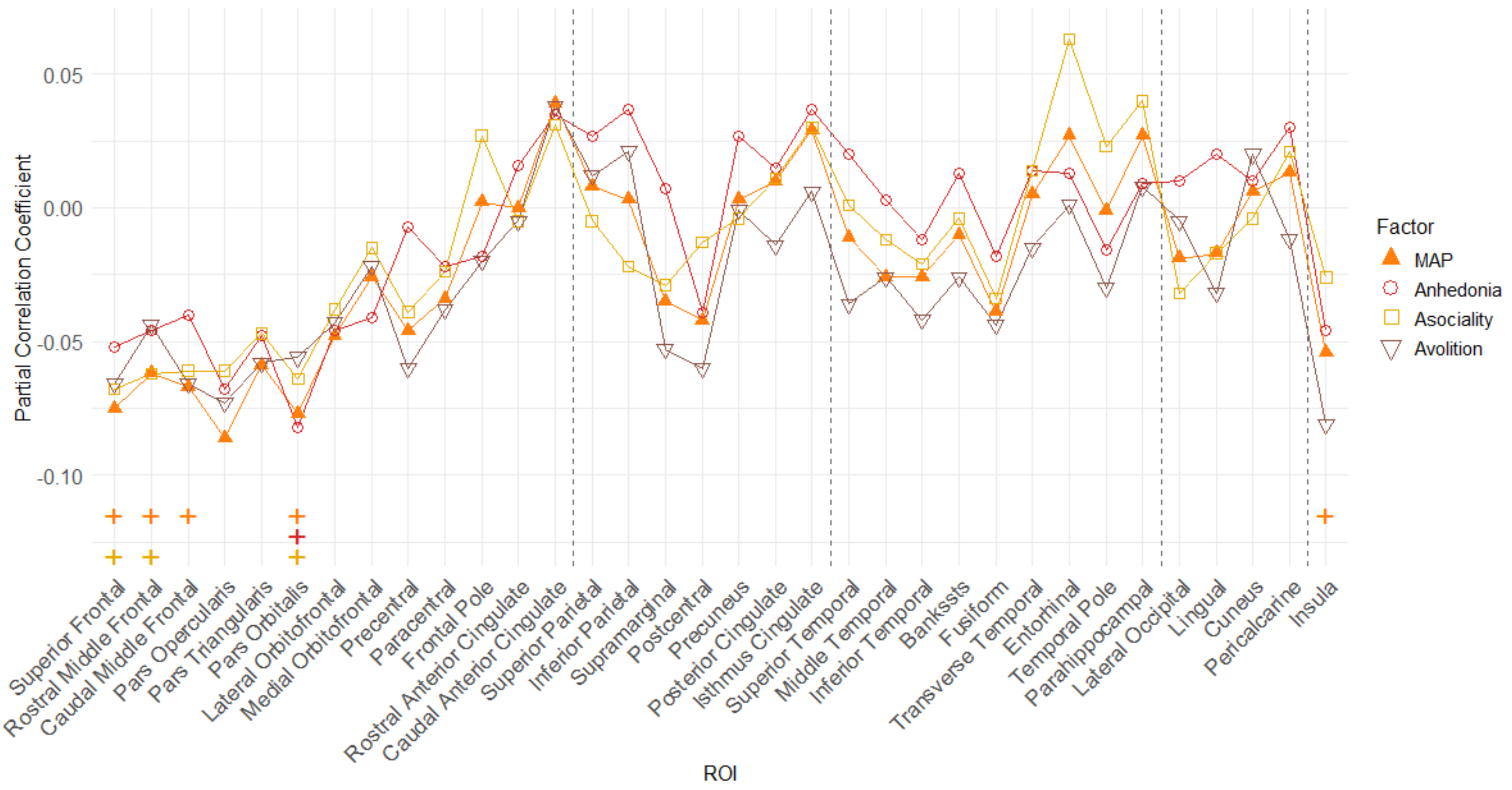

Note: FDR significant denoted with ** and nominal significance denoted with +

Supplementary Figure 1c. Pattern of Mean Correlations Between Cortical Thickness, and EXP dimensions and domains

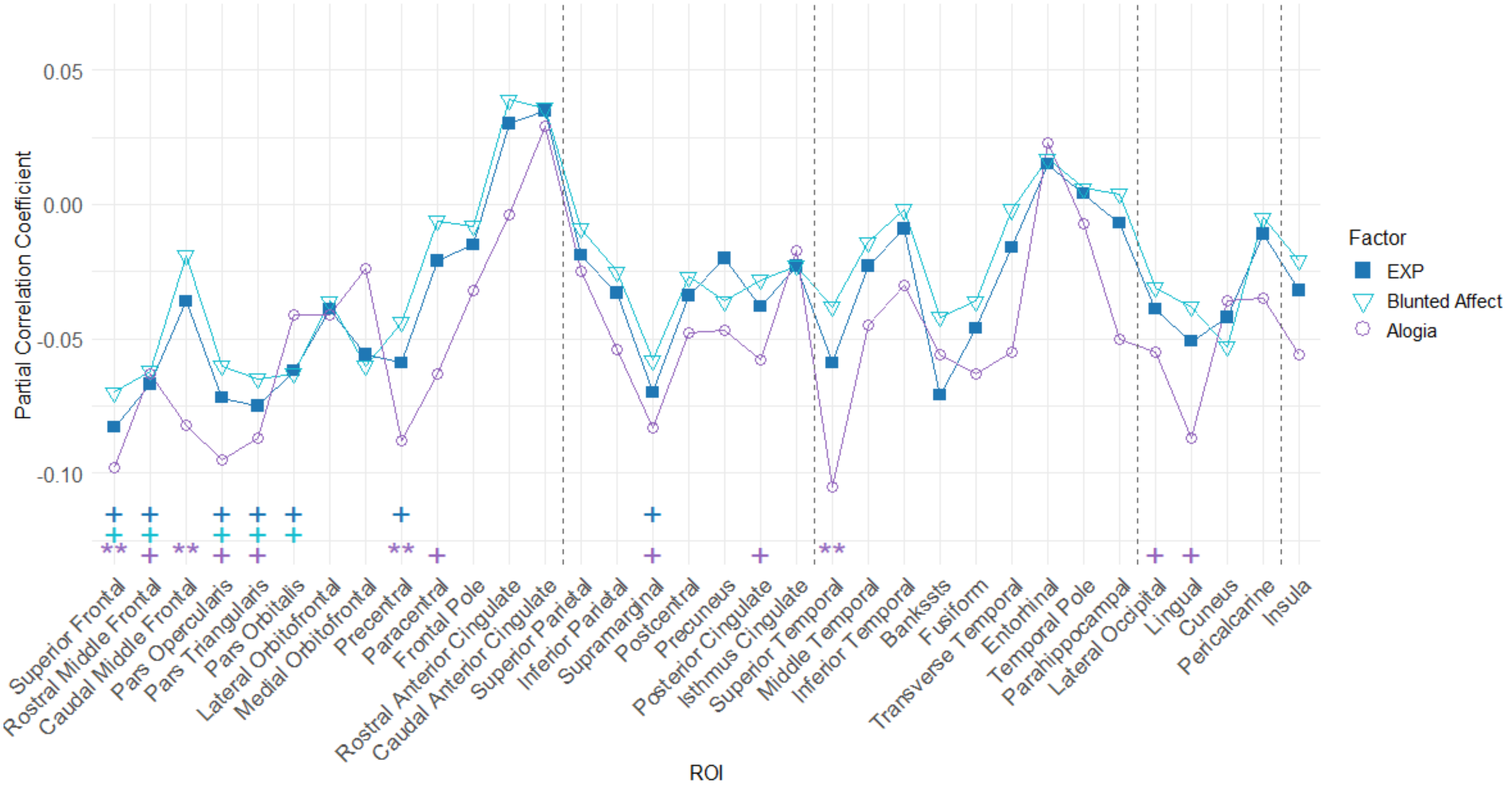

Note: FDR significant denoted with ** and nominal significance denoted with +

Supplementary Figure 2a. Pattern of Mean Correlations Between Subcortical Volumes and SANS Total, MAP and EXP

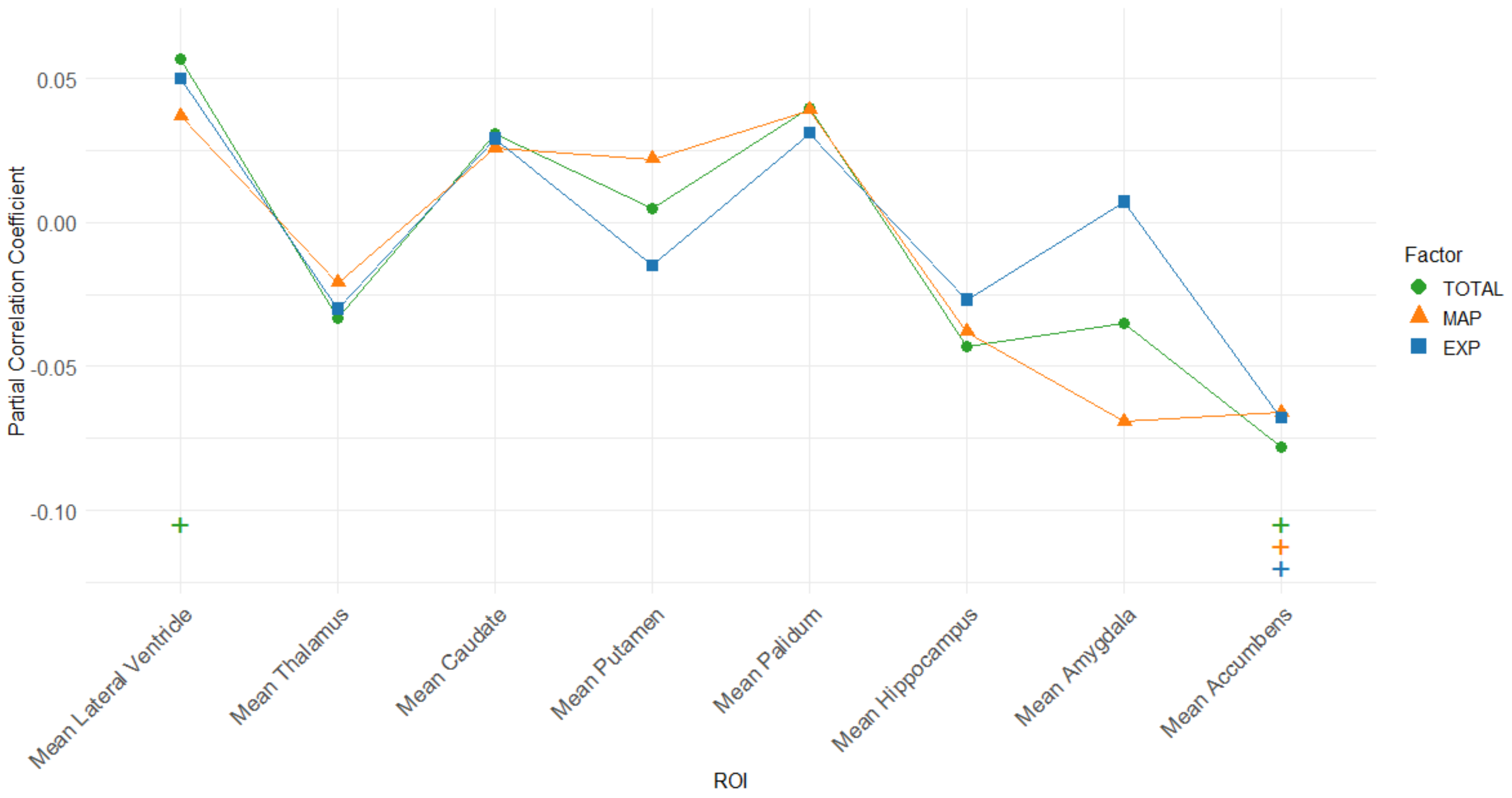

Note: Nominal significance denoted with +

Supplementary Figure 2b. Pattern of Mean Correlations Between Subcortical Volumes, and MAP dimensions and domains

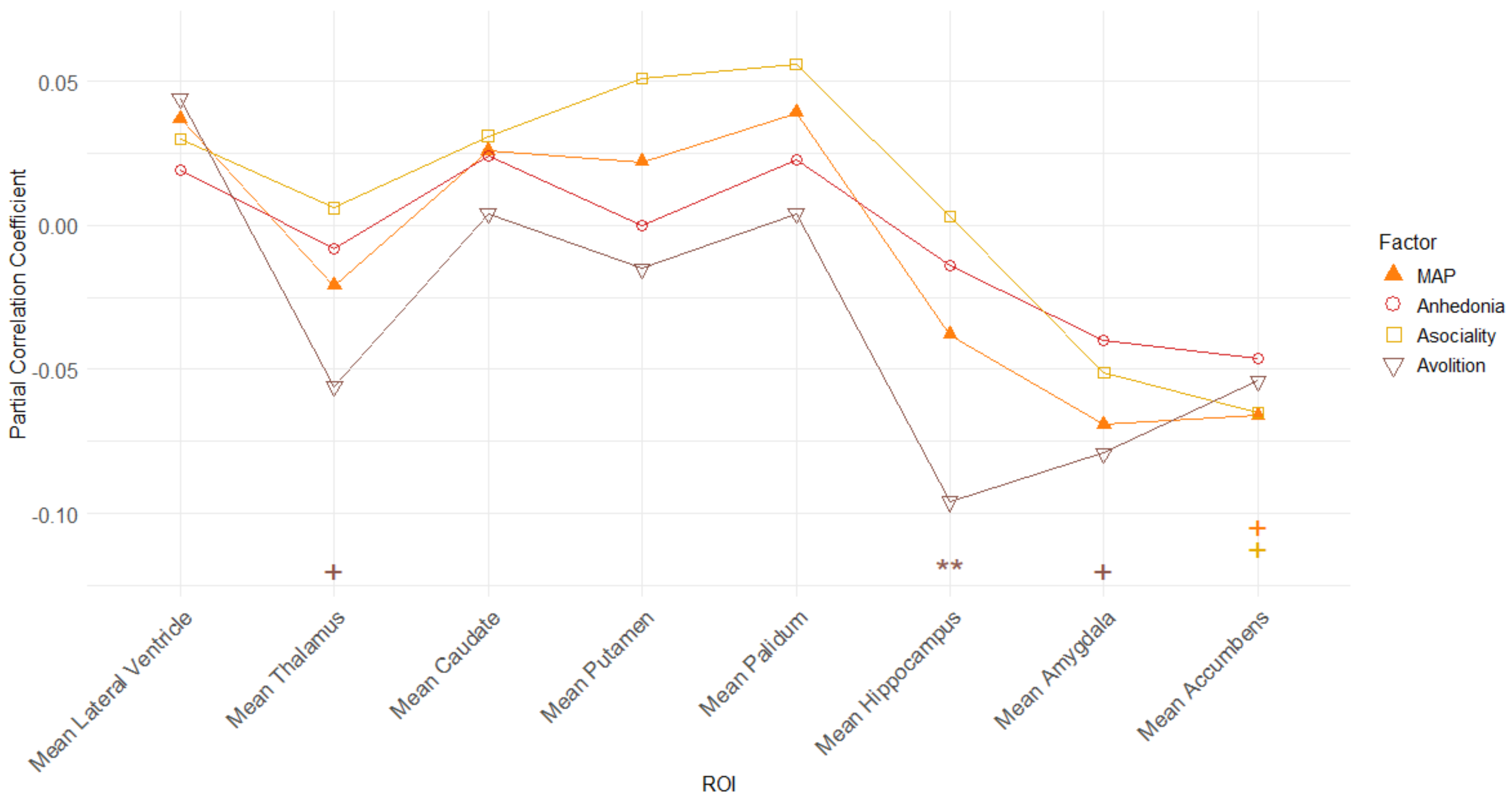

Note: FDR significant denoted with ** and nominal significance denoted with +

Supplementary Figure 2c. Pattern of Mean Correlations Between Subcortical Volumes, and EXP dimensions and domains

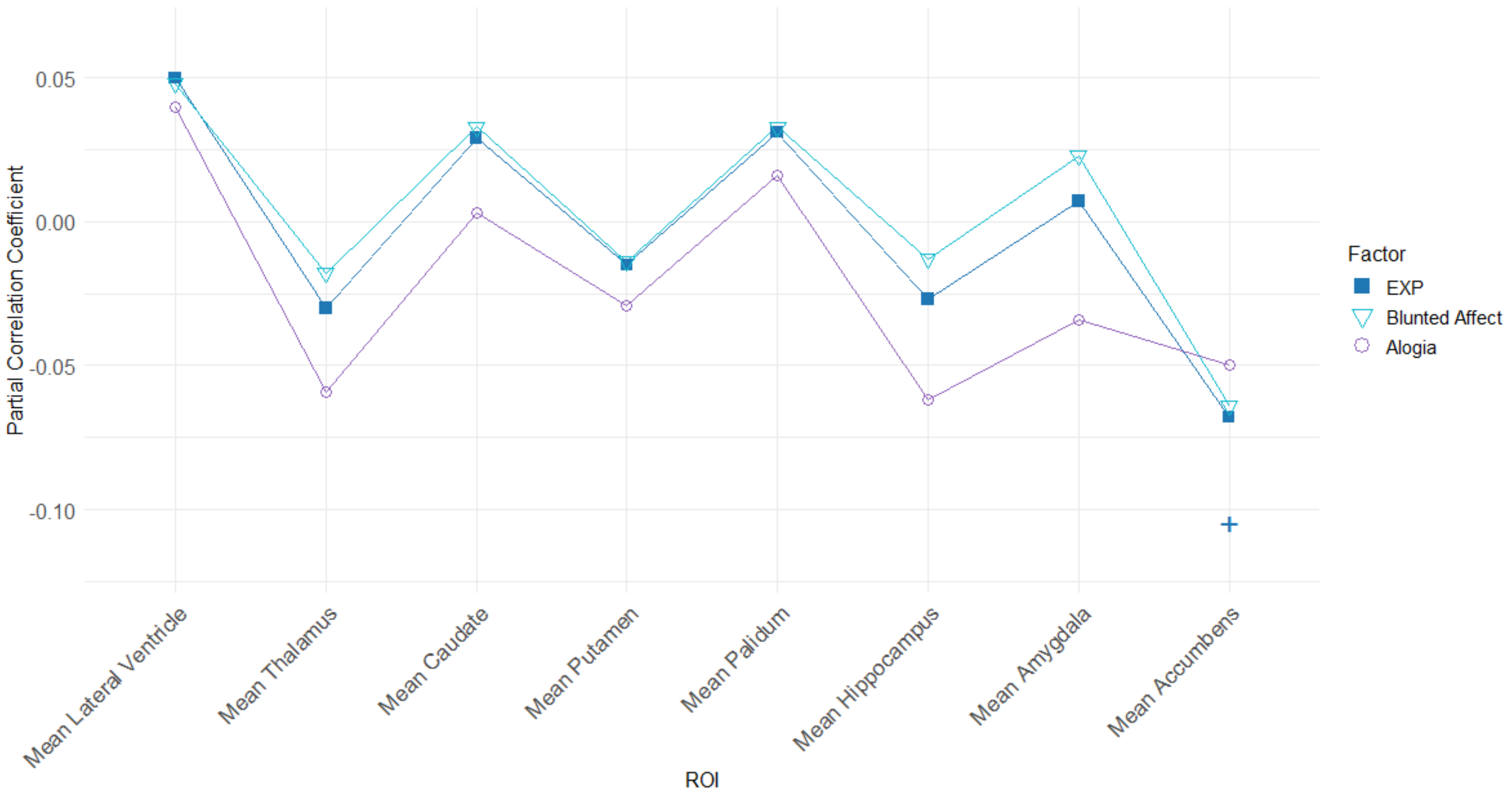

Note: Nominal significance denoted with +

Supplementary Figure 3. Traditional R and COINSTAC meta-analyses yield identical results

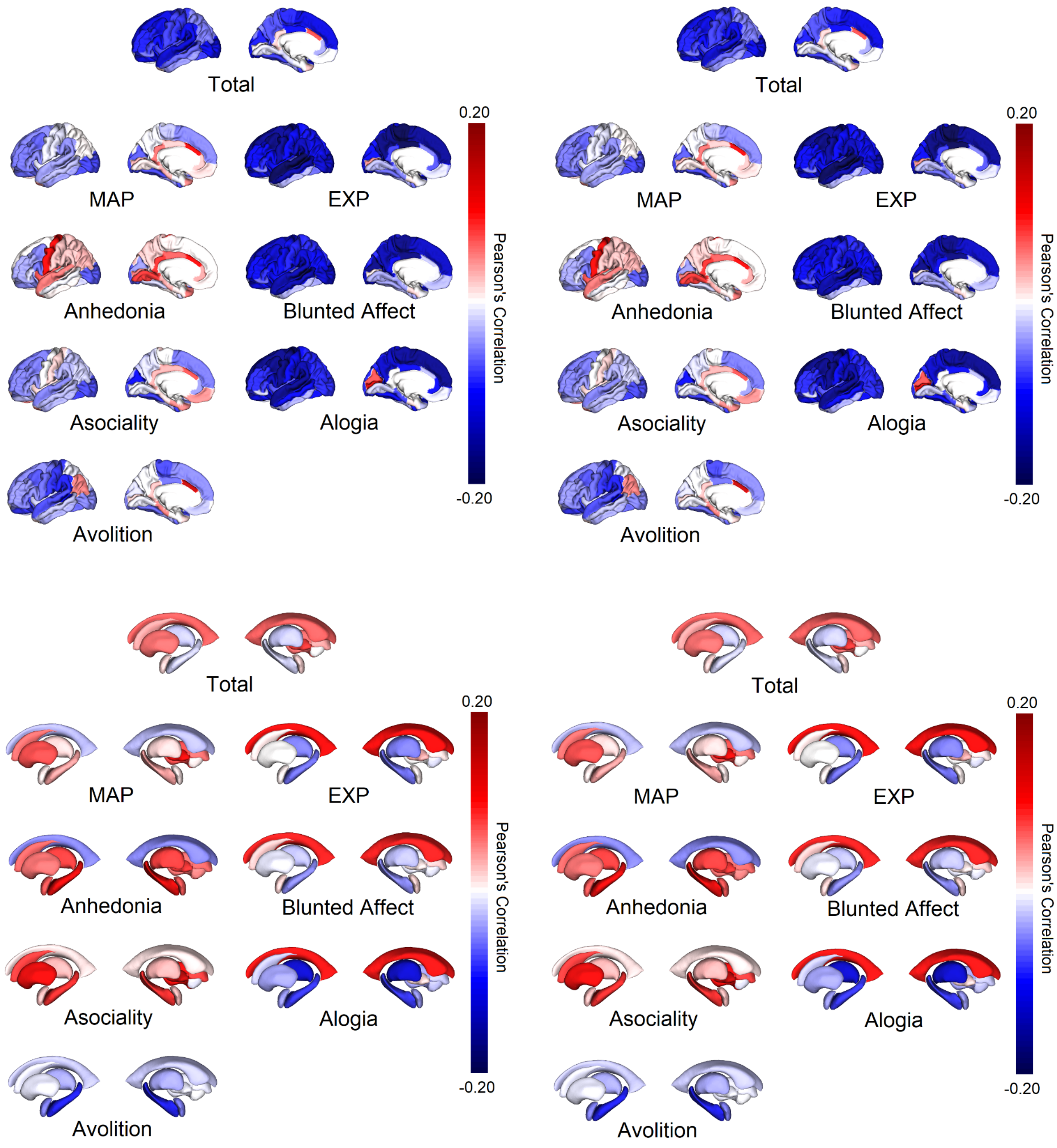

Traditional R meta-analysis (left) and COINSTAC analysis (right); analyses based on data from six sites.

Supplementary Table S1. Sample Image Acquisition and Image Processing Details

Sample	Number of Scanners	Scanner Vendor & Type	Imaging Protocols	Slice Orientation	FreeSurfer Version	Operating System	Number of subjects removed from analysis due to QC failure
ASRB	5	Siemens Avanto 1.5T	High-resolution T1-weighted structural magnetic resonance imaging (sMRI) brain scans (MPRAGE) were acquired using an optimized magnetization-prepared rapid acquisition gradient echo on 1.5 T Siemens Avanto scanners (Siemens, Erlangen, Germany) across five Australian research sites (Loughland and al., 2010). Image parameters were set to 176 slices of 1mm thickness, no gap with field-of-view 250 x 250 mm2, repetition time 1980 ms, echo time 4.3 ms, data acquisition matrix 256 x 256, with a flip matrix of 15°, resulting in a voxel size of 0.98×0.98×1.0 mm3	Sagittal	v5.1.0	Mac OSX	0

FBIRN (Phase3)	7	3T Siemens Tim Trio; 3T GE	High-resolution structural imaging scans were acquired on six 3T Siemens Tim® Trio System and one 3T General Electric Discovery MR750 scanner. MP-RAGE scan parameters for the Siemens scanner were: scan plane=sagittal, TR/TE/TI=2300/2.94/1100 ms, GRAPPA acceleration factor=2, flip angle=9°, resolution=256×256x160, FOV=220mm2, voxel size=0.86x0.86x1.2mm, and NEX=1. IR-SPGR scan parameters for the General Electric scanner were: scan plane=sagittal, TR/TE/TI=5.95/1.99/450ms , ASSET acceleration factor=2, a flip angle=12°, resolution=256×256x166, FOV=220mm2, voxel size=0.86x0.86x1.2mm, and NEX=1. All scans covered the entire brain.	Sagittal	v5.3.0	Centos 64bit	0
FIDMAG	1	1.5T GE Signa	180 axial slices; 1mm slice thickness, no gap, matrix size 512x512; 0.5x0.5x1mm3 voxel resolution; TE 4ms, TR 2000ms, flip angle 15º	Axial	v5.3.0	Linux Ubuntu	
FSLRome	1	3T Siemens Allegra	T1-weighted, 3D MDEFT, 1x1x1xmm, TE/TR =2.4/7.92 ms, flip angle=15	Sagittal	6.0dev	linux	
FOR2107-MR	1	3T Siemens Magnetom TiroTim syngo	Site Marburg: 3D T1-weighted magnetization prepared rapid acquisition gradient echo (MPRAGE); TR=1900ms, TE=2.26ms, TI=900ms, FA=9°, voxel size=1.0x1.0x1.0mm³, Acquisition Direction Sagittal, 176 slices, slice gap 0.5mm.	Sagittal	v5.3.0	Red Hat Enterprise Linux Server release 5.11 (Tikanga)	0

FOR2017-MS	1	3T Siemens PRISMA	T1-weighted magnetization prepared rapid acquisition gradient echo (MPRAGE); TR=2130ms, TE=2.28ms, TI=900ms, FA=8°, voxel size=1.0x1.0x1.0mm³, Acquisition Direction Sagittal, 192 slices, slice gap 0.5mm.	Sagittal	v5.3.0	Red Hat Enterprise Linux Server release 5.11 (Tikanga)	0
GIPSI	1	3T Philips Ingenia	3D T1-weighted TFE sequence: TR=4.7ms, TE=2ms, flip angle=8°, voxel size=1.0x0.6x0.6mm³ (no gap) and 160 axial slices	Axial	v5.3.0	Mac OSX	0
HUBIN	1	1.5T Signa HDxt	T1-weighted SPGR pulse sequence: TR=24ms, TE=6ms, FOV=240mm, acquisition matrix=256x192, 124 slices, flip angle=35 degrees, slice thickness=1.5mm	Coronal	v5.3.0	Linux	NA
IGP	1	3T Philips Achieva TX	3D MPRAGE; TR=8.9ms, TE=4.1ms, FOV=240mm, matrix 268 x 268, 200 slices, slice thickness 0.9mm (no gap)	Sagittal	v5.3.0	Mac OSX	0
MCIC	3	1.5, 3T Siemens and GE	T1 scans: TR = 2530 ms for 3 T, TR = 12 ms for 1.5 T; TE = 3.79 ms for 3 T, TE = 4.76 ms for 1.5 T; FA = 7 for 3 T, FA = 20 for 1.5 T; TI = 1100 for 3 T; Bandwidth = 181 for 3 T, Bandwidth = 110 for 1.5 T; 0.625×0.625 mm voxel size; slice thickness 1.5 mm; FOV 256×256×128 cm matrix; FOV = 16 cm (could be increased to 18 cm when needed for full brain coverage).	Coronal	v4.0.1	Linux of various flavors	5 subjects failed automated segmentation procedure due to excessive motion artifacts; 2 participants’ MRI data failed the manual inspection

NU	1	1.5T Vision	1) 3D turbo-FLASH: TR=20 ms, TE=5.4 ms, flip=30˚, ACQ=1, 256x256 matrix, 1xl mm in-plane resolution, 180 slices, slice thickness 1 mm, 13:30 min scan time and 2) 3D MPRAGE (2-4 repeats): TR=9.7 ms, TE=4 ms, flip=10˚, ACQ=1, 256x256 matrix, 1xl mm in-plane resolution, 128 slices, slice thickness 1.25 mm, 5:36 min scan time each	Axial	v5.3.0	centos6 x86_64	
SCORE	1		MPRAGE: aquisition matrix: 256×256×176, isotropic spatial resolution: 1x1x1mm3, TI=1000ms, TR=2s, TE=3.4 ms, flip angle: 8° and bandwidth of 200 Hz/pixel	Sagittal	6.0dev		
UCISZ	1	Philips 3T Achieva	T1TFE:200 sagittal slices, 320x274 matrix size, .75mm isotropic, TR = 11ms, TE =4.562ms, flip angle = 18°	Sagittal	v6.0dev	Centos 3.10.72-1.el6.elrepo.x86_64	0
UMCG	1	Philips 3T Intera	WIP T1 3D SENSE, 170 slices, 1mm slice thickness, inplane matrix 256x256, FOV: 230x170x256mm, flip angle 9 degrees)	Axial	v6.0.0	Ubuntu 16.04 LTS	0
UPENN	2	Siemens 3T	MPRAGE, TR=1810 ms, TE= 3.51 ms, TI=1100 ms, flip angle 9, FOV= 240 x 180 mm, matrix= 256 × 192, resolution = 0.9 x 0.9 mm, slices = 160, slice/skip thickness = 1 mm/0 mm	Axial	v5.3.0	Linux Red Hat Enterprise 5	0
ZURICH	1	Philips 3T	3D T1-weighted images were acquired with an ultrafast gradient echo T1-weighted sequence (TR=8.4ms, TE=3.8ms, flip angle=8°) in 160 sagittal plan slices (1mm slice thickness, no slice gap) of 240×240mm2 resulting in 1x1x1mm3voxels.	Sagittal	v6.0.0	Linux	0

Supplementary Table S2. Sample Breakdown

Sample	N	Gender M/F	Mean Age (Years)	Mean Age of Onset (Years)	Mean Duration of Illness	PANSS Total	PANSS Neg	PANSS Pos	SANS Total	SAPS Total	Mean CPZ	N No Antipsychotic Medication	N First Generation AP	N Second Generation AP	N Both First- and Second Generation AP
ASRB	263	177/86	38.6	23.6	15.0	...	...	...	18.5	...	...	44	198	12	9
FBIRN	187	141/46	38.9	21.8	17.2	58.7	14.6	15.5	19.6	19.0	372.6	0	139	20	10
FIDMAG	48	35/13	39.7	23.8	15.8	74.4	23.5	15.4	37.2	...	570.8	0	36	4	8
FOR2107-MR	37	23/14	37.2	21.2	15.9	...	...	...	18.8	13.2	403.7	6	25	3	3
FOR2107-MS	8	4/4	33.4	22.2	11.1	...	...	...	8.1	6.4	306.7	1	7	0	0
FSLRome	164	110/54	39.4	24.5	14.9	86.3	21.0	20.9	28.8	31.5	302.2	11	82	26	42
GIPSI	40	32/8	33.0	19.1	13.9	...	...	...	33.3	9.6	434.0	3	27	2	8
HUBIN	94	70/24	41.7	24.5	17.1	...	...	...	22.3	9.0	272.7	6	38	40	10
IGP	68	40/28	41.7	22.9	18.8	55.5	14.5	13.8	6.9	13.0	655.2	10	53	3	2
MCIC	148	113/35	32.9	22.8	10.2	...	...	...	23.3	22.8	533.5	8	117	10	7
NU	108	74/34	34.2	20.9	13.2	...	...	...	33.0	23.2	...	9	79	17	0
SCORE	141	98/43	25.7	24.5	1.1	...	...	...	15.1	...	207.9	104	35	2	0
UCISZ	27	22/5	42.9	25.0	17.5	60.0	16.0	15.6	22.8	13.4	...	...	...	...	...
UMCG	21	16/5	37.9	26.7	11.2	65.2	19.3	13.0	40.1	...	...	2	19	0	0
UPENN	177	105/72	38.9	20.7	17.3	...	...	...	23.7	18.3	481.6	0	65	13	5
ZURICH	60	45/15	30.5	22.2	8.4	48.6	14.5	10.7	24.9	...	494.8	3	55	0	2
Total	1591	1105/ 486	36.7	22.8	13.7	66.7	17.4	16.2	22.6	19.7	405.1	207	975	152	106

Supplementary Table S3. Higher Negative Symptom Severity in Men than Women with Schizophrenia

	**Cohen's *d***	**SE**	**95% CI**	***t*-score**	***p*-value**	***N* males**	***N* females**	**Total *N***
SANS Total	-0.233	0.064	[-0.371 - -0.096]	-3.647	0.003	1207	774	1981
EXP	-0.213	0.063	[-0.348 - -0.077]	-3.366	0.005	1269	801	2070
MAP	-0.182	0.059	[-0.309 - -0.055]	-3.082	0.008	1213	777	1990
Anhedonia	-0.131	0.045	[-0.229 - -0.034]	-2.899	0.012	1275	803	2078
Avolition	-0.139	0.052	[-0.252 - -0.027]	-2.655	0.019	1216	780	1996
Asociality	-0.217	0.063	[-0.352 - -0.082]	-3.445	0.004	1272	804	2076
Blunted Affect	-0.212	0.062	[-0.345 - -0.080]	-3.436	0.004	1273	805	2078
Alogia	-0.144	0.053	[-0.257 - -0.031]	-2.740	0.016	1274	801	2075

Note: These comparisons are based on cortical data of all individuals with schizophrenia from each site with SANS data, including ones without imaging data.

Supplementary Table S4. Sensitivity Analysis Comparing Effect Sizes of Main Analysis Against Models with the Inclusion of Medication Type and Medication Dose

	Only sex and age				WAP (medication type)				WCPZ (medication dose)				
ROI	Partial *r*	*N*	*p*	FDR	Partial *r*	*N*	*p*	FDR	Partial *r*	*N*	*p*	FDR	Factor
Left Pars Orbitalis	-0.078	1327	0.002	0.035	-0.074	1193	0.005	0.114	-0.044	747	0.306	0.960	CT SANS Total
Left Superior Frontal	-0.084	1350	0.001	0.031	-0.071	1215	0.004	0.114	-0.090	746	0.010	0.326	CT SANS Total
Right Superior Frontal	-0.096	1341	<0.001	0.03	-0.078	1206	0.002	0.114	-0.094	750	0.003	0.222	CT SANS Total
Mean Superior Frontal	-0.094	1325	<0.001	0.012	-0.077	1190	0.002	0.059	-0.097	744	0.004	0.144	CT SANS Total
Left Pars Triangularis	-0.086	1304	0.001	0.027	-0.081	1171	0.002	0.088	-0.076	746	0.024	0.329	CT Avolition
Right Caudal Middle Frontal	-0.078	1337	0.001	0.027	-0.077	1202	0.003	0.088	-0.076	749	0.019	0.329	CT Avolition
Left Rostral Middle Frontal	-0.08	1312	0.002	0.044	-0.088	1180	0.006	0.083	-0.113	745	0.001	0.023	CT Alogia
Left Superior Frontal	-0.101	1350	<0.001	0.014	-0.093	1215	0.001	0.082	-0.13	746	0.001	0.023	CT Alogia
Right Caudal Middle Frontal	-0.099	1337	0.003	0.044	-0.102	1202	0.004	0.082	-0.107	749	0.018	0.172	CT Alogia
Right Superior Frontal	-0.09	1341	0.001	0.019	-0.082	1206	0.004	0.082	-0.108	750	0.001	0.023	CT Alogia
Mean Caudal Middle Frontal	-0.082	1323	0.005	0.043	-0.092	1189	0.004	0.04	-0.094	748	0.020	0.112	CT Alogia
Mean Lingual	-0.087	1328	0.008	0.057	-0.106	1193	0.005	0.04	-0.094	729	0.058	0.198	CT Alogia
Mean Precentral	-0.088	1318	0.005	0.043	-0.09	1185	0.006	0.043	-0.102	743	0.006	0.072	CT Alogia
Mean Rostral Middle Frontal	-0.063	1263	0.013	0.065	-0.074	1131	0.014	0.081	-0.089	737	0.002	0.028	CT Alogia
Mean Superior Frontal	-0.098	1325	<0.001	0.007	-0.088	1190	0.002	0.04	-0.124	744	<0.001	0.01	CT Alogia
Mean Superior Temporal	-0.105	1132	0.002	0.034	-0.103	1001	0.003	0.04	-0.102	680	0.010	0.085	CT Alogia
Left Lateral Ventricle	0.050	1263	0.018	0.143	0.05	1211	0.001	0.017	0.064	787	0.001	0.012	SV SANS Total
Mean Lateral Ventricle	0.057	1263	0.013	0.093	0.055	1211	0.002	0.014	0.074	787	0.002	0.016	SV SANS Total
Left Hippocampus	-0.092	1339	0.001	0.007	-0.087	1196	0.005	0.038	-0.065	776	0.093	0.497	SV Avolition
Right Hippocampus	-0.096	1349	0.001	0.007	-0.093	1206	0.002	0.038	-0.084	785	0.012	0.199	SV Avolition
Mean Hippocampus	-0.096	1335	<0.001	0.004	-0.092	1193	0.003	0.021	-0.075	774	0.033	0.264	SV Avolition

Note: CT = Cortical Thickness and SV = Subcortical Volumes

Supplementary Table S5 Associations between Cortical Thickness and SANS Total

ROI	Partial *r*	SE	*t*-Score	95% CI	*I2*	*N*	*p-value*	FDR
Left Bankssts	-0.041	0.014	-1.462	[-0.100 - 0.019]	0.000	1266	0.166	0.476
Left Caudal Anterior Cingulate	*0.054*	*0.011*	*2.382*	*[0.005 - 0.103]*	*0.000*	*1362*	*0.032*	*0.235*
Left Caudal Middle Frontal	-0.035	0.014	-1.260	[-0.096 - 0.025]	0.000	1351	0.228	0.476
Left Cuneus	-0.010	0.018	-0.278	[-0.087 - 0.067]	0.000	1275	0.785	0.905
Left Entorhinal	0.000	0.011	-0.011	[-0.046 - 0.046]	0.000	1278	0.992	0.992
Left Fusiform	*-0.067*	*0.015*	*-2.175*	*[-0.133 - -0.001]*	*0.000*	*1308*	*0.047*	*0.268*
Left Inferior Parietal	0.004	0.015	0.126	[-0.059 - 0.066]	0.000	1234	0.901	0.973
Left Inferior Temporal	-0.022	0.014	-0.778	[-0.083 - 0.039]	0.000	1275	0.449	0.650
Left Isthmus Cingulate	-0.009	0.014	-0.329	[-0.067 - 0.049]	0.000	1361	0.747	0.891
Left Lateral Occipital	-0.038	0.016	-1.214	[-0.105 - 0.029]	0.000	1296	0.245	0.476
Left Lateral Orbitofrontal	-0.040	0.013	-1.520	[-0.096 - 0.016]	0.000	1364	0.151	0.469
Left Lingual	-0.039	0.015	-1.309	[-0.103 - 0.025]	0.000	1339	0.212	0.476
Left Medial Orbitofrontal	-0.039	0.016	-1.221	[-0.108 - 0.030]	0.000	1347	0.242	0.476
Left Middle Temporal	-0.029	0.014	-1.077	[-0.087 - 0.029]	0.000	1218	0.300	0.550
Left Parahippocampal	-0.009	0.015	-0.295	[-0.075 - 0.057]	0.000	1342	0.772	0.905
Left Paracentral	-0.035	0.012	-1.420	[-0.088 - 0.018]	0.000	1360	0.177	0.476
Left Pars Opercularis	*-0.094*	*0.016*	*-2.940*	*[-0.162 - -0.026]*	*0.000*	*1323*	*0.011*	*0.110*
Left Pars Orbitalis	***-0.078***	***0.010***	***-3.915***	***[-0.120 - -0.035]***	***0.000***	***1327***	***0.002***	***0.035***
Left Pars Triangularis	*-0.069*	*0.012*	*-2.889*	*[-0.121 - -0.018]*	*0.000*	*1304*	*0.012*	*0.110*
Left Pericalcarine	0.013	0.017	0.396	[-0.058 - 0.084]	0.000	1350	0.698	0.879
Left Postcentral	-0.048	0.015	-1.620	[-0.110 - 0.015]	0.000	1336	0.127	0.433
Left Posterior Cingulate	-0.028	0.015	-0.957	[-0.090 - 0.035]	0.000	1363	0.355	0.587
Left Precentral	*-0.063*	*0.014*	*-2.183*	*[-0.125 - -0.001]*	*0.000*	*1344*	*0.047*	*0.268*
Left Precuneus	-0.034	0.012	-1.415	[-0.087 - 0.018]	0.000	1337	0.179	0.476
Left Rostral Anterior Cingulate	0.029	0.011	1.262	[-0.020 - 0.078]	0.000	1347	0.228	0.476
Left Rostral Middle Frontal	*-0.061*	*0.011*	*-2.847*	*[-0.107 - -0.015]*	*0.000*	*1312*	*0.013*	*0.110*
Left Superior Frontal	***-0.084***	***0.010***	***-4.180***	***[-0.127 - -0.041]***	***0.000***	***1350***	***0.001***	***0.031***
Left Superior Parietal	-0.002	0.019	-0.044	[-0.081 - 0.078]	23.374	1283	0.966	0.986
Left Superior Temporal	-0.055	0.014	-1.955	[-0.114 - 0.005]	0.000	1197	0.071	0.283
Left Supramarginal	*-0.056*	*0.012*	*-2.340*	*[-0.106 - -0.005]*	*0.000*	*1216*	*0.035*	*0.235*
Left Frontal Pole	-0.018	0.011	-0.800	[-0.065 - 0.030]	0.000	1368	0.437	0.646
Left Temporal Pole	-0.009	0.011	-0.437	[-0.054 - 0.036]	0.000	1329	0.669	0.858
Left Transverse Temporal	0.007	0.014	0.255	[-0.052 - 0.065]	0.000	1362	0.802	0.909
Left Insula	-0.040	0.015	-1.355	[-0.103 - 0.023]	0.000	1365	0.197	0.476
Right Bankssts	-0.040	0.023	-0.857	[-0.139 - 0.060]	57.461	1317	0.406	0.613
Right Caudal Anterior Cingulate	0.003	0.016	0.088	[-0.067 - 0.073]	0.000	1361	0.931	0.986
Right Caudal Middle Frontal	*-0.085*	*0.013*	*-3.331*	*[-0.139 - -0.030]*	*0.000*	*1337*	*0.005*	*0.084*
Right Cuneus	-0.021	0.015	-0.724	[-0.084 - 0.042]	0.000	1316	0.481	0.674
Right Entorhinal	0.032	0.012	1.277	[-0.021 - 0.084]	0.000	1164	0.222	0.476
Right Fusiform	-0.034	0.014	-1.188	[-0.095 - 0.027]	0.000	1281	0.255	0.481
Right Inferior Parietal	-0.028	0.019	-0.716	[-0.110 - 0.055]	0.000	1226	0.486	0.674
Right Inferior Temporal	-0.033	0.018	-0.923	[-0.111 - 0.044]	13.384	1283	0.371	0.587
Right Isthmus Cingulate	0.008	0.012	0.341	[-0.044 - 0.060]	0.000	1351	0.739	0.891
Right Lateral Occipital	-0.033	0.017	-0.953	[-0.106 - 0.041]	0.000	1287	0.357	0.587
Right Lateral Orbitofrontal	-0.057	0.016	-1.816	[-0.124 - 0.010]	0.000	1333	0.091	0.343
Right Lingual	-0.029	0.014	-1.060	[-0.089 - 0.030]	0.000	1350	0.307	0.550
Right Medial Orbitofrontal	-0.047	0.018	-1.347	[-0.122 - 0.028]	7.462	1316	0.199	0.476
Right Middle Temporal	-0.028	0.020	-0.698	[-0.112 - 0.057]	23.117	1255	0.497	0.676

ROI	Partial *r*	SE	*t*-Score	95% CI	*I2*	*N*	*p-value*	FDR
Right Parahippocampal	0.022	0.012	0.935	[-0.028 - 0.072]	0.000	1322	0.365	0.587
Right Paracentral	-0.029	0.010	-1.516	[-0.070 - 0.012]	0.000	1361	0.152	0.469
Right Pars Opercularis	-0.063	0.016	-1.980	[-0.131 - 0.005]	0.000	1305	0.068	0.283
Right Pars Orbitalis	-0.035	0.013	-1.366	[-0.090 - 0.020]	0.000	1336	0.193	0.476
Right Pars Triangularis	-0.055	0.013	-2.066	[-0.113 - 0.002]	0.000	1305	0.058	0.283
Right Pericalcarine	-0.016	0.014	-0.579	[-0.077 - 0.045]	0.000	1350	0.572	0.762
Right Postcentral	-0.035	0.014	-1.241	[-0.096 - 0.026]	0.000	1342	0.235	0.476
Right Posterior Cingulate	-0.012	0.013	-0.481	[-0.067 - 0.043]	0.000	1364	0.638	0.834
Right Precentral	-0.040	0.010	-2.025	[-0.083 - 0.002]	0.000	1333	0.062	0.283
Right Precuneus	0.001	0.012	0.051	[-0.052 - 0.054]	0.000	1333	0.960	0.986
Right Rostral Anterior Cingulate	-0.013	0.020	-0.335	[-0.098 - 0.072]	46.962	1327	0.742	0.891
Right Rostral Middle Frontal	*-0.065*	*0.011*	*-3.029*	*[-0.111 - -0.019]*	*0.000*	*1302*	*0.009*	*0.110*
Right Superior Frontal	***-0.096***	***0.010***	***-4.570***	***[-0.141 - -0.051]***	***0.000***	***1341***	***<0.001***	***0.030***
Right Superior Parietal	-0.004	0.016	-0.136	[-0.073 - 0.064]	10.016	1314	0.893	0.973
Right Superior Temporal	-0.034	0.017	-0.996	[-0.108 - 0.039]	0.000	1234	0.336	0.586
Right Supramarginal	-0.069	0.020	-1.692	[-0.156 - 0.019]	44.337	1223	0.113	0.403
Right Frontal Pole	-0.004	0.013	-0.153	[-0.059 - 0.051]	0.000	1365	0.881	0.973
Right Temporal Pole	-0.001	0.008	-0.037	[-0.034 - 0.033]	0.000	1225	0.971	0.986
Right Transverse Temporal	-0.022	0.012	-0.891	[-0.074 - 0.031]	0.000	1363	0.388	0.600
Right Insula	-0.060	0.015	-2.022	[-0.123 - 0.004]	1.166	1363	0.063	0.283
Left Thickness	*-0.055*	*0.013*	*-2.181*	*[-0.108 - -0.001]*	*0.000*	*1370*	*0.047*	*1.000*
Right Thickness	*-0.056*	*0.012*	*-2.251*	*[-0.109 - -0.003]*	*0.000*	*1370*	*0.041*	*1.000*
Left Surface Area	-0.049	0.017	-1.422	[-0.122 - 0.025]	5.082	1370	0.177	1.000
Right Surface Area	-0.041	0.017	-1.246	[-0.112 - 0.030]	0.000	1370	0.233	1.000
Mean Bankssts	-0.043	0.018	-1.197	[-0.119 - 0.034]	0.000	1230	0.251	0.474
Mean Caudal Anterior Cingulate	0.043	0.013	1.589	[-0.015 - 0.100]	0.000	1354	0.134	0.326
Mean Caudal Middle Frontal	*-0.064*	*0.014*	*-2.334*	*[-0.122 - -0.005]*	*0.000*	*1323*	*0.035*	*0.170*
Mean Cuneus	-0.029	0.021	-0.697	[-0.117 - 0.060]	47.276	1243	0.497	0.705
Mean Entorhinal	0.017	0.012	0.733	[-0.034 - 0.069]	0.000	1137	0.476	0.705
Mean Fusiform	-0.055	0.015	-1.843	[-0.119 - 0.009]	0.000	1240	0.087	0.326
Mean Inferior Parietal	-0.021	0.017	-0.597	[-0.094 - 0.053]	0.000	1160	0.560	0.733
Mean Inferior Temporal	-0.025	0.017	-0.745	[-0.098 - 0.047]	0.000	1227	0.468	0.705
Mean Isthmus Cingulate	0.000	0.013	0.009	[-0.055 - 0.056]	0.000	1345	0.993	0.993
Mean Lateral Occipital	-0.038	0.018	-1.080	[-0.114 - 0.038]	11.541	1237	0.299	0.534
Mean Lateral Orbitofrontal	-0.048	0.015	-1.637	[-0.110 - 0.015]	0.000	1327	0.124	0.326
Mean Lingual	-0.044	0.015	-1.482	[-0.107 - 0.020]	0.000	1328	0.160	0.349
Mean Medial Orbitofrontal	-0.050	0.017	-1.430	[-0.124 - 0.025]	0.000	1298	0.175	0.349
Mean Middle Temporal	-0.029	0.019	-0.782	[-0.108 - 0.051]	8.438	1162	0.447	0.705
Mean Parahippocampal	0.008	0.014	0.269	[-0.053 - 0.068]	0.000	1305	0.792	0.899
Mean Paracentral	-0.033	0.011	-1.467	[-0.080 - 0.015]	0.000	1352	0.164	0.349
Mean Pars Opercularis	*-0.089*	*0.017*	*-2.578*	*[-0.162 - -0.015]*	*0.000*	*1273*	*0.022*	*0.124*
Mean Pars Orbitalis	*-0.078*	*0.011*	*-3.554*	*[-0.125 - -0.031]*	*0.000*	*1301*	*0.003*	*0.052*
Mean Pars Triangularis	*-0.078*	*0.014*	*-2.864*	*[-0.137 - -0.020]*	*0.000*	*1256*	*0.013*	*0.085*
Mean Pericalcarine	-0.002	0.016	-0.062	[-0.069 - 0.065]	0.000	1340	0.951	0.980
Mean Postcentral	-0.047	0.014	-1.659	[-0.108 - 0.014]	0.000	1316	0.119	0.326
Mean Posterior Cingulate	-0.020	0.015	-0.696	[-0.083 - 0.042]	0.000	1358	0.498	0.705
Mean Precentral	*-0.066*	*0.011*	*-2.964*	*[-0.114 - -0.018]*	*0.000*	*1318*	*0.010*	*0.085*
Mean Precuneus	-0.016	0.013	-0.633	[-0.072 - 0.039]	0.000	1311	0.537	0.730
Mean Rostral Anterior Cingulate	0.015	0.016	0.491	[-0.052 - 0.083]	7.899	1309	0.631	0.795
Mean Rostral Middle Frontal	*-0.071*	*0.010*	*-3.365*	*[-0.115 - -0.026]*	*0.000*	*1263*	*0.005*	*0.052*
Mean Superior Frontal	***-0.094***	***0.010***	***-4.686***	***[-0.136 - -0.051]***	***0.000***	***1325***	***<0.001***	***0.012***

ROI	Partial *r*	SE	*t*-Score	95% CI	*I2*	*N*	*p-value*	FDR
Mean Superior Parietal	-0.005	0.018	-0.145	[-0.084 - 0.073]	20.122	1248	0.887	0.950
Mean Superior Temporal	-0.049	0.015	-1.632	[-0.112 - 0.015]	0.000	1132	0.125	0.326
Mean Supramarginal	*-0.068*	*0.015*	*-2.209*	*[-0.133 - -0.002]*	*0.000*	*1147*	*0.044*	*0.188*
Mean Frontal Pole	-0.007	0.013	-0.267	[-0.060 - 0.047]	0.000	1363	0.793	0.899
Mean Temporal Pole	-0.002	0.008	-0.136	[-0.035 - 0.031]	0.000	1206	0.894	0.950
Mean Transverse Temporal	-0.009	0.012	-0.379	[-0.063 - 0.044]	0.000	1356	0.710	0.862
Mean Insula	-0.053	0.015	-1.780	[-0.117 - 0.011]	0.000	1358	0.097	0.326

Note: nominally significant results are italicized and FDR significant results are italicized and bolded

Supplementary Table S6 Associations between Cortical Thickness and SANS MAP

ROI	Partial *r*	SE	*t*-Score	95% CI	*I2*	*N*	*p-value*	FDR
Left Bankssts	-0.010	0.014	-0.338	[-0.070 - 0.051]	0.000	1266	0.740	0.912
Left Caudal Anterior Cingulate	0.049	0.015	1.602	[-0.016 - 0.113]	0.000	1362	0.131	0.526
Left Caudal Middle Frontal	-0.049	0.015	-1.671	[-0.111 - 0.014]	0.000	1351	0.117	0.497
Left Cuneus	-0.009	0.015	-0.305	[-0.072 - 0.054]	0.000	1275	0.765	0.912
Left Entorhinal	-0.001	0.011	-0.056	[-0.047 - 0.044]	0.000	1278	0.956	0.970
Left Fusiform	-0.049	0.013	-1.835	[-0.105 - 0.008]	0.000	1308	0.088	0.446
Left Inferior Parietal	0.025	0.014	0.918	[-0.033 - 0.083]	0.000	1234	0.374	0.714
Left Inferior Temporal	-0.023	0.013	-0.886	[-0.080 - 0.033]	0.000	1275	0.391	0.714
Left Isthmus Cingulate	0.017	0.016	0.531	[-0.053 - 0.087]	0.000	1361	0.604	0.838
Left Lateral Occipital	-0.011	0.017	-0.334	[-0.084 - 0.062]	0.000	1296	0.744	0.912
Left Lateral Orbitofrontal	-0.042	0.010	-2.009	[-0.087 - 0.003]	0.000	1364	0.064	0.397
Left Lingual	-0.017	0.014	-0.602	[-0.078 - 0.044]	0.000	1339	0.557	0.838
Left Medial Orbitofrontal	-0.014	0.015	-0.459	[-0.080 - 0.052]	0.000	1347	0.653	0.888
Left Middle Temporal	-0.014	0.014	-0.531	[-0.073 - 0.044]	0.000	1218	0.604	0.838
Left Parahippocampal	0.011	0.013	0.409	[-0.046 - 0.067]	0.000	1342	0.688	0.912
Left Paracentral	-0.043	0.015	-1.489	[-0.106 - 0.019]	0.000	1360	0.159	0.583
Left Pars Opercularis	*-0.092*	*0.019*	*-2.451*	*[-0.171 - -0.012]*	*8.474*	*1323*	*0.028*	*0.230*
Left Pars Orbitalis	*-0.063*	*0.010*	*-3.020*	*[-0.108 - -0.018]*	*0.000*	*1327*	*0.009*	*0.167*
Left Pars Triangularis	*-0.062*	*0.012*	*-2.660*	*[-0.112 - -0.012]*	*0.000*	*1304*	*0.019*	*0.230*
Left Pericalcarine	-0.008	0.025	-0.164	[-0.115 - 0.099]	63.999	1350	0.872	0.926
Left Postcentral	-0.037	0.017	-1.079	[-0.109 - 0.036]	0.000	1336	0.299	0.714
Left Posterior Cingulate	0.015	0.013	0.575	[-0.040 - 0.070]	0.000	1363	0.574	0.838
Left Precentral	-0.052	0.019	-1.351	[-0.133 - 0.030]	34.545	1344	0.198	0.674
Left Precuneus	-0.019	0.010	-0.948	[-0.063 - 0.025]	0.000	1337	0.359	0.714
Left Rostral Anterior Cingulate	0.004	0.012	0.165	[-0.049 - 0.058]	0.000	1347	0.871	0.926
Left Rostral Middle Frontal	*-0.051*	*0.010*	*-2.408*	*[-0.095 - -0.006]*	*0.000*	*1312*	*0.030*	*0.230*
Left Superior Frontal	*-0.059*	*0.012*	*-2.348*	*[-0.112 - -0.005]*	*0.000*	*1350*	*0.034*	*0.232*
Left Superior Parietal	0.010	0.016	0.326	[-0.058 - 0.079]	0.000	1283	0.749	0.912
Left Superior Temporal	-0.022	0.016	-0.703	[-0.090 - 0.046]	0.000	1197	0.494	0.781
Left Supramarginal	-0.022	0.013	-0.868	[-0.076 - 0.032]	0.000	1216	0.400	0.714
Left Frontal Pole	-0.017	0.012	-0.721	[-0.066 - 0.033]	0.000	1368	0.483	0.781
Left Temporal Pole	-0.024	0.013	-0.932	[-0.078 - 0.031]	0.000	1329	0.367	0.714
Left Transverse Temporal	0.024	0.013	0.932	[-0.032 - 0.081]	0.000	1362	0.367	0.714
Left Insula	-0.039	0.011	-1.809	[-0.085 - 0.007]	0.000	1365	0.092	0.446
Right Bankssts	-0.003	0.015	-0.119	[-0.066 - 0.059]	0.000	1317	0.907	0.935
Right Caudal Anterior Cingulate	0.003	0.012	0.140	[-0.046 - 0.053]	0.000	1361	0.891	0.932

ROI	Partial *r*	SE	*t*-Score	95% CI	*I2*	*N*	*p-value*	FDR
Right Caudal Middle Frontal	*-0.075*	*0.012*	*-2.986*	*[-0.128 - -0.021]*	*0.000*	*1337*	*0.010*	*0.167*
Right Cuneus	0.020	0.012	0.826	[-0.032 - 0.073]	0.000	1316	0.423	0.719
Right Entorhinal	0.033	0.010	1.703	[-0.009 - 0.075]	0.000	1164	0.111	0.497
Right Fusiform	-0.034	0.014	-1.246	[-0.093 - 0.025]	0.000	1281	0.233	0.692
Right Inferior Parietal	-0.009	0.019	-0.242	[-0.090 - 0.072]	0.000	1226	0.812	0.921
Right Inferior Temporal	-0.029	0.018	-0.775	[-0.107 - 0.050]	23.828	1283	0.451	0.748
Right Isthmus Cingulate	0.032	0.012	1.275	[-0.022 - 0.085]	0.000	1351	0.223	0.692
Right Lateral Occipital	-0.029	0.017	-0.865	[-0.101 - 0.043]	0.000	1287	0.402	0.714
Right Lateral Orbitofrontal	-0.055	0.014	-1.922	[-0.115 - 0.006]	0.000	1333	0.075	0.426
Right Lingual	-0.008	0.014	-0.284	[-0.068 - 0.052]	0.000	1350	0.781	0.915
Right Medial Orbitofrontal	-0.032	0.018	-0.910	[-0.107 - 0.043]	0.000	1316	0.378	0.714
Right Middle Temporal	-0.039	0.023	-0.851	[-0.135 - 0.059]	46.262	1255	0.409	0.714
Right Parahippocampal	0.033	0.014	1.216	[-0.025 - 0.091]	0.000	1322	0.244	0.692
Right Paracentral	-0.023	0.013	-0.882	[-0.077 - 0.032]	0.000	1361	0.393	0.714
Right Pars Opercularis	-0.061	0.021	-1.472	[-0.148 - 0.028]	45.063	1305	0.163	0.583
Right Pars Orbitalis	*-0.050*	*0.010*	*-2.436*	*[-0.093 - -0.006]*	*0.000*	*1336*	*0.029*	*0.230*
Right Pars Triangularis	-0.036	0.015	-1.219	[-0.100 - 0.027]	0.000	1305	0.243	0.692
Right Pericalcarine	0.005	0.014	0.186	[-0.054 - 0.064]	0.000	1350	0.855	0.926
Right Postcentral	-0.020	0.019	-0.541	[-0.101 - 0.061]	32.594	1342	0.597	0.838
Right Posterior Cingulate	0.000	0.011	0.003	[-0.046 - 0.047]	0.000	1364	0.998	0.998
Right Precentral	-0.025	0.013	-0.980	[-0.080 - 0.030]	0.000	1333	0.344	0.714
Right Precuneus	0.023	0.010	1.156	[-0.019 - 0.065]	0.000	1333	0.267	0.714
Right Rostral Anterior Cingulate	-0.011	0.016	-0.360	[-0.078 - 0.056]	1.721	1327	0.724	0.912
Right Rostral Middle Frontal	*-0.062*	*0.010*	*-3.137*	*[-0.105 - -0.020]*	*0.000*	*1302*	*0.007*	*0.167*
Right Superior Frontal	*-0.083*	*0.013*	*-3.301*	*[-0.136 - -0.029]*	*0.000*	*1341*	*0.005*	*0.167*
Right Superior Parietal	0.008	0.016	0.254	[-0.060 - 0.076]	0.000	1314	0.803	0.921
Right Superior Temporal	-0.005	0.014	-0.167	[-0.065 - 0.055]	0.000	1234	0.870	0.926
Right Supramarginal	-0.047	0.025	-0.930	[-0.154 - 0.061]	66.345	1223	0.368	0.714
Right Frontal Pole	0.009	0.015	0.312	[-0.054 - 0.072]	0.000	1365	0.760	0.912
Right Temporal Pole	0.021	0.011	0.971	[-0.025 - 0.067]	0.000	1225	0.348	0.714
Right Transverse Temporal	-0.013	0.011	-0.588	[-0.062 - 0.035]	0.000	1363	0.566	0.838
Right Insula	*-0.063*	*0.012*	*-2.552*	*[-0.116 - -0.010]*	*0.000*	*1363*	*0.023*	*0.230*
Left Thickness	-0.037	0.013	-1.390	[-0.094 - 0.020]	0.000	1370	0.186	1.000
Right Thickness	-0.037	0.014	-1.322	[-0.096 - 0.023]	0.000	1370	0.207	1.000
Left Surface Area	-0.056	0.017	-1.606	[-0.130 - 0.019]	0.000	1370	0.130	1.000
Right Surface Area	-0.054	0.016	-1.660	[-0.123 - 0.016]	0.000	1370	0.119	1.000
Mean Bankssts	-0.010	0.014	-0.382	[-0.069 - 0.048]	0.000	1230	0.708	0.956
Mean Caudal Anterior Cingulate	0.039	0.013	1.443	[-0.019 - 0.097]	0.000	1354	0.171	0.576
Mean Caudal Middle Frontal	*-0.067*	*0.014*	*-2.388*	*[-0.126 - -0.007]*	*0.000*	*1323*	*0.032*	*0.269*
Mean Cuneus	0.006	0.015	0.213	[-0.057 - 0.069]	0.000	1243	0.835	0.987
Mean Entorhinal	0.027	0.013	1.040	[-0.028 - 0.082]	0.000	1137	0.316	0.722
Mean Fusiform	-0.039	0.014	-1.390	[-0.100 - 0.021]	0.000	1240	0.186	0.576
Mean Inferior Parietal	0.003	0.017	0.092	[-0.068 - 0.074]	0.000	1160	0.928	0.987
Mean Inferior Temporal	-0.026	0.017	-0.770	[-0.098 - 0.046]	0.000	1227	0.454	0.812
Mean Isthmus Cingulate	0.029	0.015	0.988	[-0.034 - 0.092]	0.000	1345	0.340	0.722
Mean Lateral Occipital	-0.019	0.018	-0.518	[-0.097 - 0.060]	0.000	1237	0.613	0.947
Mean Lateral Orbitofrontal	-0.048	0.012	-2.032	[-0.099 - 0.003]	0.000	1327	0.062	0.269
Mean Lingual	-0.017	0.015	-0.586	[-0.080 - 0.046]	0.000	1328	0.567	0.918
Mean Medial Orbitofrontal	-0.026	0.017	-0.781	[-0.099 - 0.046]	0.000	1298	0.448	0.812
Mean Middle Temporal	-0.026	0.021	-0.622	[-0.114 - 0.063]	29.114	1162	0.544	0.918
Mean Parahippocampal	0.027	0.014	0.941	[-0.034 - 0.087]	0.000	1305	0.363	0.725

ROI	Partial *r*	SE	*t*-Score	95% CI	*I2*	*N*	*p-value*	FDR
Mean Paracentral	-0.034	0.014	-1.215	[-0.092 - 0.026]	0.000	1352	0.244	0.660
Mean Pars Opercularis	-0.086	0.021	-2.017	[-0.177 - 0.005]	39.194	1273	0.063	0.269
Mean Pars Orbitalis	*-0.077*	*0.010*	*-3.894*	*[-0.118 - -0.034]*	*0.000*	*1301*	*0.002*	*0.055*
Mean Pars Triangularis	-0.059	0.014	-2.144	[-0.118 - 0.000]	0.000	1256	0.050	0.269
Mean Pericalcarine	0.013	0.018	0.374	[-0.064 - 0.090]	0.000	1340	0.714	0.956
Mean Postcentral	-0.042	0.017	-1.194	[-0.116 - 0.033]	0.000	1316	0.252	0.660
Mean Posterior Cingulate	0.010	0.012	0.417	[-0.042 - 0.062]	0.000	1358	0.683	0.956
Mean Precentral	-0.046	0.015	-1.569	[-0.109 - 0.017]	0.000	1318	0.139	0.525
Mean Precuneus	0.003	0.010	0.142	[-0.039 - 0.044]	0.000	1311	0.889	0.987
Mean Rostral Anterior Cingulate	0.000	0.015	-0.016	[-0.064 - 0.063]	0.000	1309	0.987	0.987
Mean Rostral Middle Frontal	*-0.062*	*0.010*	*-3.111*	*[-0.104 - -0.019]*	*0.000*	*1263*	*0.008*	*0.087*
Mean Superior Frontal	*-0.075*	*0.012*	*-3.133*	*[-0.126 - -0.024]*	*0.000*	*1325*	*0.007*	*0.087*
Mean Superior Parietal	0.008	0.016	0.247	[-0.062 - 0.078]	0.000	1248	0.808	0.987
Mean Superior Temporal	-0.011	0.015	-0.351	[-0.075 - 0.054]	0.000	1132	0.731	0.956
Mean Supramarginal	-0.035	0.017	-0.991	[-0.109 - 0.040]	0.000	1147	0.338	0.722
Mean Frontal Pole	0.002	0.014	0.077	[-0.060 - 0.064]	0.000	1363	0.940	0.987
Mean Temporal Pole	-0.001	0.010	-0.047	[-0.042 - 0.040]	0.000	1206	0.963	0.987
Mean Transverse Temporal	0.005	0.012	0.196	[-0.047 - 0.057]	0.000	1356	0.848	0.987
Mean Insula	*-0.054*	*0.012*	*-2.245*	*[-0.106 - -0.002]*	*0.000*	*1358*	*0.041*	*0.269*

Note: nominally significant results are italicized and FDR significant results are italicized and bolded

Supplementary Table S7 Associations between Cortical Thickness and SANS Anhedonia (Factor 1)

ROI	Partial *r*	SE	*t*-Score	95% CI	*I2*	*N*	*p-value*	FDR
Left Bankssts	0.014	0.014	0.485	[-0.048 - 0.076]	0.000	1266	0.635	0.948
Left Caudal Anterior Cingulate	0.056	0.014	1.968	[-0.005 - 0.116]	0.000	1362	0.069	0.507
Left Caudal Middle Frontal	-0.024	0.013	-0.896	[-0.081 - 0.033]	0.000	1351	0.385	0.784
Left Cuneus	-0.003	0.011	-0.120	[-0.051 - 0.045]	0.000	1275	0.906	0.948
Left Entorhinal	-0.013	0.007	-0.969	[-0.042 - 0.016]	0.000	1278	0.349	0.784
Left Fusiform	-0.031	0.007	-2.101	[-0.062 - 0.001]	0.000	1308	0.054	0.507
Left Inferior Parietal	0.045	0.014	1.570	[-0.017 - 0.107]	0.000	1234	0.139	0.551
Left Inferior Temporal	-0.025	0.013	-0.964	[-0.080 - 0.030]	0.000	1275	0.351	0.784
Left Isthmus Cingulate	0.016	0.017	0.462	[-0.058 - 0.089]	0.000	1361	0.651	0.948
Left Lateral Occipital	0.010	0.016	0.302	[-0.059 - 0.079]	0.000	1296	0.767	0.948
Left Lateral Orbitofrontal	*-0.046*	*0.010*	*-2.356*	*[-0.087 - -0.004]*	*0.000*	*1364*	*0.034*	*0.507*
Left Lingual	0.013	0.012	0.544	[-0.039 - 0.065]	0.000	1339	0.595	0.948
Left Medial Orbitofrontal	-0.032	0.015	-1.071	[-0.095 - 0.032]	0.000	1347	0.302	0.784
Left Middle Temporal	-0.005	0.012	-0.195	[-0.057 - 0.047]	0.000	1218	0.848	0.948
Left Parahippocampal	-0.006	0.013	-0.253	[-0.061 - 0.049]	0.000	1342	0.804	0.948
Left Paracentral	-0.006	0.022	-0.127	[-0.098 - 0.087]	47.282	1360	0.901	0.948
Left Pars Opercularis	*-0.088*	*0.015*	*-3.015*	*[-0.150 - -0.026]*	*0.000*	*1323*	*0.009*	*0.290*
Left Pars Orbitalis	*-0.070*	*0.009*	*-3.802*	*[-0.109 - -0.031]*	*0.000*	*1327*	*0.002*	*0.132*
Left Pars Triangularis	*-0.057*	*0.010*	*-2.853*	*[-0.100 - -0.014]*	*0.000*	*1304*	*0.013*	*0.290*
Left Pericalcarine	0.009	0.020	0.224	[-0.078 - 0.096]	42.040	1350	0.826	0.948
Left Postcentral	-0.035	0.019	-0.933	[-0.114 - 0.045]	0.000	1336	0.367	0.784
Left Posterior Cingulate	0.012	0.016	0.370	[-0.056 - 0.079]	0.000	1363	0.717	0.948
Left Precentral	-0.006	0.016	-0.186	[-0.075 - 0.063]	0.000	1344	0.855	0.948
Left Precuneus	0.001	0.012	0.051	[-0.051 - 0.053]	0.000	1337	0.960	0.974
Left Rostral Anterior Cingulate	0.015	0.015	0.475	[-0.052 - 0.081]	7.451	1347	0.642	0.948
Left Rostral Middle Frontal	-0.044	0.011	-2.063	[-0.090 - 0.002]	0.000	1312	0.058	0.507
Left Superior Frontal	-0.041	0.013	-1.589	[-0.095 - 0.014]	0.000	1350	0.134	0.551

ROI	Partial *r*	SE	*t*-Score	95% CI	*I2*	*N*	*p-value*	FDR
Left Superior Parietal	0.015	0.016	0.480	[-0.053 - 0.083]	0.000	1283	0.639	0.948
Left Superior Temporal	0.009	0.013	0.351	[-0.047 - 0.065]	0.000	1197	0.731	0.948
Left Supramarginal	0.010	0.015	0.332	[-0.055 - 0.075]	0.026	1216	0.745	0.948
Left Frontal Pole	-0.008	0.013	-0.300	[-0.063 - 0.048]	0.000	1368	0.768	0.948
Left Temporal Pole	-0.041	0.014	-1.507	[-0.099 - 0.017]	0.000	1329	0.154	0.551
Left Transverse Temporal	0.020	0.007	1.351	[-0.011 - 0.051]	0.000	1362	0.198	0.673
Left Insula	-0.043	0.014	-1.517	[-0.104 - 0.018]	0.000	1365	0.152	0.551
Right Bankssts	0.014	0.012	0.555	[-0.039 - 0.066]	0.000	1317	0.588	0.948
Right Caudal Anterior Cingulate	-0.011	0.011	-0.519	[-0.058 - 0.035]	0.000	1361	0.612	0.948
Right Caudal Middle Frontal	-0.052	0.014	-1.863	[-0.112 - 0.008]	0.000	1337	0.084	0.507
Right Cuneus	0.023	0.013	0.915	[-0.031 - 0.078]	0.000	1316	0.375	0.784
Right Entorhinal	0.017	0.010	0.840	[-0.027 - 0.062]	0.000	1164	0.415	0.784
Right Fusiform	-0.018	0.008	-1.138	[-0.053 - 0.016]	0.000	1281	0.274	0.784
Right Inferior Parietal	0.023	0.017	0.709	[-0.047 - 0.094]	0.000	1226	0.490	0.877
Right Inferior Temporal	0.007	0.014	0.237	[-0.054 - 0.067]	0.000	1283	0.816	0.948
Right Isthmus Cingulate	0.048	0.014	1.737	[-0.011 - 0.108]	0.000	1351	0.104	0.507
Right Lateral Occipital	0.002	0.016	0.064	[-0.068 - 0.072]	11.604	1287	0.950	0.974
Right Lateral Orbitofrontal	-0.051	0.014	-1.860	[-0.109 - 0.008]	0.537	1333	0.084	0.507
Right Lingual	0.028	0.015	0.922	[-0.037 - 0.093]	0.000	1350	0.372	0.784
Right Medial Orbitofrontal	-0.045	0.017	-1.314	[-0.117 - 0.028]	28.664	1316	0.210	0.680
Right Middle Temporal	0.004	0.017	0.120	[-0.069 - 0.078]	0.000	1255	0.906	0.948
Right Parahippocampal	0.021	0.014	0.768	[-0.037 - 0.079]	0.000	1322	0.455	0.837
Right Paracentral	-0.011	0.016	-0.326	[-0.080 - 0.059]	0.000	1361	0.749	0.948
Right Pars Opercularis	-0.046	0.023	-1.000	[-0.145 - 0.053]	60.735	1305	0.334	0.784
Right Pars Orbitalis	*-0.055*	*0.012*	*-2.243*	*[-0.107 - -0.002]*	*0.000*	*1336*	*0.042*	*0.507*
Right Pars Triangularis	-0.023	0.018	-0.651	[-0.100 - 0.054]	0.000	1305	0.526	0.917
Right Pericalcarine	0.028	0.013	1.065	[-0.029 - 0.085]	0.000	1350	0.305	0.784
Right Postcentral	-0.029	0.017	-0.857	[-0.100 - 0.043]	9.654	1342	0.406	0.784
Right Posterior Cingulate	0.008	0.015	0.269	[-0.057 - 0.074]	0.000	1364	0.792	0.948
Right Precentral	0.004	0.016	0.142	[-0.062 - 0.071]	0.000	1333	0.889	0.948
Right Precuneus	0.043	0.012	1.758	[-0.009 - 0.094]	0.000	1333	0.101	0.507
Right Rostral Anterior Cingulate	0.005	0.016	0.144	[-0.063 - 0.072]	0.000	1327	0.887	0.948
Right Rostral Middle Frontal	-0.043	0.013	-1.646	[-0.099 - 0.013]	0.000	1302	0.122	0.551
Right Superior Frontal	-0.058	0.015	-1.902	[-0.124 - 0.007]	0.000	1341	0.078	0.507
Right Superior Parietal	0.033	0.016	1.045	[-0.035 - 0.102]	0.000	1314	0.314	0.784
Right Superior Temporal	0.018	0.010	0.863	[-0.027 - 0.062]	0.000	1234	0.403	0.784
Right Supramarginal	0.000	0.016	0.003	[-0.069 - 0.070]	0.000	1223	0.997	0.997
Right Frontal Pole	-0.035	0.014	-1.233	[-0.097 - 0.026]	0.000	1365	0.238	0.735
Right Temporal Pole	0.007	0.011	0.326	[-0.039 - 0.053]	0.000	1225	0.749	0.948
Right Transverse Temporal	0.005	0.012	0.219	[-0.047 - 0.058]	0.000	1363	0.830	0.948
Right Insula	-0.046	0.013	-1.802	[-0.100 - 0.009]	0.000	1363	0.093	0.507
Left Thickness	-0.018	0.013	-0.675	[-0.073 - 0.038]	0.000	1370	0.511	1.000
Right Thickness	-0.012	0.014	-0.410	[-0.072 - 0.049]	0.000	1370	0.688	1.000
Left Surface Area	-0.046	0.015	-1.576	[-0.108 - 0.017]	0.000	1370	0.137	1.000
Right Surface Area	-0.041	0.013	-1.524	[-0.099 - 0.017]	0.000	1370	0.150	1.000
Mean Bankssts	0.013	0.013	0.496	[-0.042 - 0.067]	0.000	1230	0.628	0.788
Mean Caudal Anterior Cingulate	0.035	0.011	1.578	[-0.013 - 0.083]	0.000	1354	0.137	0.583
Mean Caudal Middle Frontal	-0.040	0.014	-1.456	[-0.099 - 0.019]	0.000	1323	0.167	0.624
Mean Cuneus	0.010	0.012	0.432	[-0.041 - 0.061]	0.000	1243	0.672	0.788
Mean Entorhinal	0.013	0.010	0.613	[-0.031 - 0.057]	0.000	1137	0.550	0.779
Mean Fusiform	-0.018	0.007	-1.387	[-0.047 - 0.010]	0.000	1240	0.187	0.624

ROI	Partial *r*	SE	*t*-Score	95% CI	*I2*	*N*	*p-value*	FDR
Mean Inferior Parietal	0.037	0.015	1.222	[-0.028 - 0.103]	0.000	1160	0.242	0.670
Mean Inferior Temporal	-0.012	0.014	-0.439	[-0.071 - 0.047]	0.000	1227	0.667	0.788
Mean Isthmus Cingulate	0.037	0.016	1.133	[-0.033 - 0.105]	0.000	1345	0.276	0.670
Mean Lateral Occipital	0.010	0.017	0.304	[-0.063 - 0.083]	0.000	1237	0.766	0.840
Mean Lateral Orbitofrontal	-0.046	0.011	-2.069	[-0.094 - 0.002]	0.000	1327	0.057	0.583
Mean Lingual	0.020	0.014	0.720	[-0.040 - 0.081]	0.000	1328	0.484	0.779
Mean Medial Orbitofrontal	-0.041	0.015	-1.340	[-0.107 - 0.025]	0.000	1298	0.202	0.624
Mean Middle Temporal	0.003	0.015	0.112	[-0.063 - 0.070]	0.000	1162	0.912	0.912
Mean Parahippocampal	0.009	0.012	0.370	[-0.043 - 0.060]	0.000	1305	0.717	0.813
Mean Paracentral	-0.022	0.017	-0.652	[-0.093 - 0.050]	0.000	1352	0.525	0.779
Mean Pars Opercularis	-0.068	0.020	-1.727	[-0.152 - 0.017]	26.002	1273	0.106	0.583
Mean Pars Orbitalis	*-0.082*	*0.012*	*-3.512*	*[-0.131 - -0.032]*	*0.000*	*1301*	*0.003*	*0.117*
Mean Pars Triangularis	-0.048	0.015	-1.657	[-0.111 - 0.014]	0.000	1256	0.120	0.583
Mean Pericalcarine	0.030	0.016	0.971	[-0.036 - 0.097]	0.000	1340	0.348	0.740
Mean Postcentral	-0.039	0.018	-1.066	[-0.117 - 0.040]	0.000	1316	0.305	0.690
Mean Posterior Cingulate	0.015	0.016	0.461	[-0.054 - 0.083]	0.000	1358	0.652	0.788
Mean Precentral	-0.007	0.016	-0.232	[-0.075 - 0.060]	0.000	1318	0.820	0.869
Mean Precuneus	0.027	0.012	1.165	[-0.023 - 0.077]	0.000	1311	0.263	0.670
Mean Rostral Anterior Cingulate	0.016	0.015	0.544	[-0.048 - 0.081]	0.000	1309	0.595	0.788
Mean Rostral Middle Frontal	-0.046	0.012	-1.832	[-0.099 - 0.008]	0.000	1263	0.088	0.583
Mean Superior Frontal	-0.052	0.013	-1.946	[-0.108 - 0.005]	0.000	1325	0.072	0.583
Mean Superior Parietal	0.027	0.016	0.844	[-0.042 - 0.096]	0.000	1248	0.413	0.779
Mean Superior Temporal	0.020	0.012	0.793	[-0.034 - 0.073]	0.000	1132	0.441	0.779
Mean Supramarginal	0.007	0.017	0.201	[-0.066 - 0.080]	0.000	1147	0.843	0.869
Mean Frontal Pole	-0.018	0.015	-0.635	[-0.081 - 0.044]	0.000	1363	0.536	0.779
Mean Temporal Pole	-0.016	0.011	-0.754	[-0.062 - 0.030]	0.000	1206	0.463	0.779
Mean Transverse Temporal	0.014	0.009	0.772	[-0.025 - 0.054]	0.000	1356	0.453	0.779
Mean Insula	-0.046	0.014	-1.576	[-0.107 - 0.016]	0.000	1358	0.137	0.583

Note: nominally significant results are italicized and FDR significant results are italicized and bolded

Supplementary Table S8 Associations between Cortical Thickness and SANS Asociality (Factor 2)

ROI	Partial *r*	SE	*t*-Score	95% CI	*I2*	*N*	*p-value*	FDR
Left Bankssts	0.005	0.013	0.171	[-0.052 - 0.061]	0.000	1266	0.866	0.944
Left Caudal Anterior Cingulate	0.047	0.015	1.562	[-0.017 - 0.110]	0.000	1362	0.141	0.742
Left Caudal Middle Frontal	-0.047	0.015	-1.556	[-0.111 - 0.018]	0.000	1351	0.142	0.742
Left Cuneus	-0.016	0.016	-0.511	[-0.083 - 0.051]	0.000	1275	0.617	0.933
Left Entorhinal	0.005	0.014	0.177	[-0.054 - 0.064]	0.000	1278	0.862	0.944
Left Fusiform	-0.039	0.015	-1.344	[-0.101 - 0.023]	0.000	1308	0.200	0.813
Left Inferior Parietal	-0.004	0.012	-0.166	[-0.055 - 0.047]	0.000	1234	0.870	0.944
Left Inferior Temporal	-0.013	0.010	-0.606	[-0.057 - 0.032]	0.000	1275	0.554	0.925
Left Isthmus Cingulate	0.022	0.017	0.634	[-0.052 - 0.096]	0.000	1361	0.536	0.925
Left Lateral Occipital	-0.021	0.017	-0.600	[-0.094 - 0.053]	0.000	1296	0.558	0.925
Left Lateral Orbitofrontal	-0.027	0.012	-1.118	[-0.080 - 0.025]	0.000	1364	0.282	0.813
Left Lingual	-0.018	0.016	-0.574	[-0.086 - 0.050]	0.000	1339	0.575	0.927
Left Medial Orbitofrontal	-0.005	0.015	-0.157	[-0.067 - 0.058]	0.000	1347	0.877	0.944
Left Middle Temporal	-0.007	0.013	-0.276	[-0.060 - 0.047]	0.000	1218	0.787	0.944
Left Parahippocampal	0.021	0.013	0.802	[-0.036 - 0.078]	0.000	1342	0.436	0.905
Left Paracentral	-0.029	0.012	-1.191	[-0.080 - 0.023]	0.000	1360	0.253	0.813
Left Pars Opercularis	-0.075	0.018	-2.024	[-0.154 - 0.004]	26.678	1323	0.062	0.609
Left Pars Orbitalis	-0.053	0.013	-2.022	[-0.108 - 0.003]	0.000	1327	0.063	0.609

ROI	Partial *r*	SE	*t*-Score	95% CI	*I2*	*N*	*p-value*	FDR
Left Pars Triangularis	-0.032	0.014	-1.097	[-0.094 - 0.030]	0.000	1304	0.291	0.813
Left Pericalcarine	-0.016	0.027	-0.288	[-0.130 - 0.100]	70.860	1350	0.777	0.944
Left Postcentral	-0.013	0.016	-0.388	[-0.082 - 0.057]	0.000	1336	0.704	0.944
Left Posterior Cingulate	0.014	0.008	0.922	[-0.019 - 0.048]	0.000	1363	0.372	0.845
Left Precentral	-0.038	0.017	-1.132	[-0.109 - 0.034]	0.000	1344	0.277	0.813
Left Precuneus	-0.021	0.012	-0.920	[-0.071 - 0.028]	0.000	1337	0.373	0.845
Left Rostral Anterior Cingulate	0.000	0.014	0.002	[-0.058 - 0.058]	0.000	1347	0.998	0.998
Left Rostral Middle Frontal	-0.044	0.012	-1.874	[-0.094 - 0.006]	0.000	1312	0.082	0.619
Left Superior Frontal	-0.050	0.013	-1.928	[-0.106 - 0.006]	0.000	1350	0.074	0.619
Left Superior Parietal	0.007	0.015	0.237	[-0.056 - 0.070]	0.000	1283	0.816	0.944
Left Superior Temporal	-0.012	0.016	-0.381	[-0.080 - 0.056]	0.000	1197	0.709	0.944
Left Supramarginal	-0.022	0.011	-0.988	[-0.069 - 0.026]	0.000	1216	0.340	0.845
Left Frontal Pole	0.001	0.013	0.041	[-0.054 - 0.056]	0.000	1368	0.968	0.982
Left Temporal Pole	0.019	0.015	0.655	[-0.043 - 0.081]	0.000	1329	0.523	0.925
Left Transverse Temporal	0.032	0.015	1.093	[-0.031 - 0.094]	0.000	1362	0.293	0.813
Left Insula	-0.004	0.010	-0.210	[-0.049 - 0.040]	0.000	1365	0.837	0.944
Right Bankssts	-0.009	0.014	-0.302	[-0.071 - 0.053]	0.000	1317	0.767	0.944
Right Caudal Anterior Cingulate	-0.007	0.012	-0.302	[-0.058 - 0.044]	0.000	1361	0.767	0.944
Right Caudal Middle Frontal	-0.061	0.015	-2.063	[-0.124 - 0.002]	0.000	1337	0.058	0.609
Right Cuneus	0.012	0.015	0.378	[-0.054 - 0.078]	0.000	1316	0.711	0.944
Right Entorhinal	0.055	0.016	1.733	[-0.013 - 0.122]	0.000	1164	0.105	0.649
Right Fusiform	-0.028	0.015	-0.953	[-0.091 - 0.035]	0.000	1281	0.357	0.845
Right Inferior Parietal	-0.020	0.018	-0.557	[-0.096 - 0.056]	0.000	1226	0.586	0.927
Right Inferior Temporal	-0.023	0.017	-0.680	[-0.095 - 0.049]	0.000	1283	0.507	0.925
Right Isthmus Cingulate	0.026	0.012	1.079	[-0.025 - 0.077]	0.000	1351	0.299	0.813
Right Lateral Occipital	-0.043	0.017	-1.248	[-0.118 - 0.031]	0.000	1287	0.232	0.813
Right Lateral Orbitofrontal	-0.050	0.014	-1.776	[-0.110 - 0.010]	0.000	1333	0.097	0.649
Right Lingual	-0.011	0.013	-0.400	[-0.068 - 0.046]	0.000	1350	0.695	0.944
Right Medial Orbitofrontal	-0.015	0.015	-0.490	[-0.081 - 0.051]	0.000	1316	0.631	0.933
Right Middle Temporal	-0.034	0.022	-0.772	[-0.127 - 0.060]	48.321	1255	0.453	0.906
Right Parahippocampal	0.047	0.016	1.462	[-0.022 - 0.114]	0.410	1322	0.166	0.806
Right Paracentral	-0.018	0.011	-0.797	[-0.065 - 0.030]	0.000	1361	0.439	0.905
Right Pars Opercularis	-0.040	0.017	-1.150	[-0.115 - 0.035]	0.000	1305	0.270	0.813
Right Pars Orbitalis	-0.042	0.010	-2.064	[-0.086 - 0.002]	0.000	1336	0.058	0.609
Right Pars Triangularis	-0.041	0.015	-1.406	[-0.103 - 0.021]	0.000	1305	0.182	0.813
Right Pericalcarine	0.020	0.015	0.650	[-0.045 - 0.085]	0.000	1350	0.526	0.925
Right Postcentral	-0.004	0.016	-0.129	[-0.074 - 0.066]	0.000	1342	0.899	0.944
Right Posterior Cingulate	0.004	0.009	0.220	[-0.033 - 0.041]	0.000	1364	0.829	0.944
Right Precentral	-0.023	0.014	-0.816	[-0.083 - 0.037]	0.000	1333	0.428	0.905
Right Precuneus	0.014	0.010	0.686	[-0.030 - 0.057]	0.000	1333	0.504	0.925
Right Rostral Anterior Cingulate	-0.008	0.015	-0.255	[-0.073 - 0.058]	0.000	1327	0.802	0.944
Right Rostral Middle Frontal	*-0.066*	*0.011*	*-3.068*	*[-0.112 - -0.020]*	*0.000*	*1302*	*0.008*	*0.538*
Right Superior Frontal	*-0.080*	*0.014*	*-2.745*	*[-0.141 - -0.017]*	*5.541*	*1341*	*0.016*	*0.538*
Right Superior Parietal	-0.008	0.017	-0.218	[-0.082 - 0.067]	0.000	1314	0.831	0.944
Right Superior Temporal	0.003	0.016	0.107	[-0.064 - 0.070]	0.000	1234	0.916	0.944
Right Supramarginal	-0.019	0.020	-0.495	[-0.103 - 0.064]	0.000	1223	0.629	0.933
Right Frontal Pole	0.031	0.015	1.009	[-0.034 - 0.095]	0.000	1365	0.330	0.845
Right Temporal Pole	0.032	0.012	1.320	[-0.020 - 0.084]	0.000	1225	0.208	0.813
Right Transverse Temporal	-0.002	0.009	-0.117	[-0.041 - 0.037]	0.000	1363	0.908	0.944
Right Insula	*-0.045*	*0.009*	*-2.511*	*[-0.084 - -0.007]*	*0.000*	*1363*	*0.025*	*0.565*
Left Thickness	-0.029	0.012	-1.164	[-0.083 - 0.025]	0.000	1370	0.264	1.000
Right Thickness	-0.033	0.013	-1.231	[-0.089 - 0.024]	0.000	1370	0.239	1.000

ROI	Partial *r*	SE	*t*-Score	95% CI	*I2*	*N*	*p-value*	FDR
Left Surface Area	-0.046	0.023	-0.994	[-0.144 - 0.053]	55.705	1370	0.337	1.000
Right Surface Area	-0.043	0.022	-0.963	[-0.138 - 0.053]	53.299	1370	0.352	1.000
Mean Bankssts	-0.004	0.013	-0.168	[-0.058 - 0.050]	0.000	1230	0.869	0.934
Mean Caudal Anterior Cingulate	0.031	0.012	1.288	[-0.021 - 0.083]	0.000	1354	0.219	0.620
Mean Caudal Middle Frontal	-0.061	0.016	-1.855	[-0.130 - 0.010]	10.602	1323	0.085	0.620
Mean Cuneus	-0.004	0.017	-0.108	[-0.076 - 0.069]	0.000	1243	0.915	0.943
Mean Entorhinal	0.063	0.020	1.592	[-0.022 - 0.147]	37.808	1137	0.134	0.620
Mean Fusiform	-0.034	0.015	-1.108	[-0.099 - 0.032]	2.753	1240	0.286	0.699
Mean Inferior Parietal	-0.022	0.015	-0.714	[-0.088 - 0.044]	0.000	1160	0.487	0.770
Mean Inferior Temporal	-0.021	0.014	-0.741	[-0.081 - 0.040]	0.000	1227	0.471	0.770
Mean Isthmus Cingulate	0.030	0.015	0.992	[-0.034 - 0.094]	0.000	1345	0.338	0.722
Mean Lateral Occipital	-0.032	0.018	-0.879	[-0.110 - 0.046]	0.000	1237	0.394	0.722
Mean Lateral Orbitofrontal	-0.038	0.014	-1.377	[-0.098 - 0.021]	0.000	1327	0.190	0.620
Mean Lingual	-0.017	0.015	-0.581	[-0.081 - 0.047]	0.000	1328	0.570	0.808
Mean Medial Orbitofrontal	-0.015	0.015	-0.480	[-0.079 - 0.050]	0.000	1298	0.639	0.835
Mean Middle Temporal	-0.012	0.018	-0.337	[-0.090 - 0.065]	12.345	1162	0.741	0.900
Mean Parahippocampal	0.040	0.016	1.294	[-0.027 - 0.107]	0.000	1305	0.217	0.620
Mean Paracentral	-0.024	0.011	-1.104	[-0.071 - 0.023]	0.000	1352	0.288	0.699
Mean Pars Opercularis	-0.061	0.018	-1.692	[-0.137 - 0.016]	0.000	1273	0.113	0.620
Mean Pars Orbitalis	*-0.064*	*0.011*	*-2.888*	*[-0.111 - -0.017]*	*0.000*	*1301*	*0.012*	*0.204*
Mean Pars Triangularis	-0.047	0.015	-1.597	[-0.110 - 0.016]	0.000	1256	0.133	0.620
Mean Pericalcarine	0.021	0.019	0.544	[-0.061 - 0.102]	0.000	1340	0.595	0.809
Mean Postcentral	-0.013	0.017	-0.377	[-0.084 - 0.059]	0.000	1316	0.712	0.897
Mean Posterior Cingulate	0.011	0.008	0.696	[-0.024 - 0.046]	0.000	1358	0.498	0.770
Mean Precentral	-0.039	0.015	-1.309	[-0.104 - 0.025]	0.000	1318	0.212	0.620
Mean Precuneus	-0.004	0.011	-0.197	[-0.051 - 0.042]	0.000	1311	0.846	0.934
Mean Rostral Anterior Cingulate	-0.005	0.016	-0.160	[-0.072 - 0.062]	0.000	1309	0.875	0.934
Mean Rostral Middle Frontal	*-0.062*	*0.011*	*-2.885*	*[-0.109 - -0.016]*	*0.000*	*1263*	*0.012*	*0.204*
Mean Superior Frontal	*-0.068*	*0.014*	*-2.483*	*[-0.126 - -0.009]*	*0.000*	*1325*	*0.026*	*0.298*
Mean Superior Parietal	-0.005	0.016	-0.155	[-0.074 - 0.064]	0.000	1248	0.879	0.934
Mean Superior Temporal	0.001	0.016	0.026	[-0.067 - 0.069]	0.000	1132	0.980	0.980
Mean Supramarginal	-0.029	0.017	-0.867	[-0.102 - 0.043]	0.000	1147	0.401	0.722
Mean Frontal Pole	0.027	0.016	0.862	[-0.040 - 0.094]	0.000	1363	0.403	0.722
Mean Temporal Pole	0.023	0.012	0.942	[-0.030 - 0.077]	0.000	1206	0.362	0.722
Mean Transverse Temporal	0.014	0.012	0.611	[-0.036 - 0.065]	0.000	1356	0.551	0.808
Mean Insula	-0.026	0.010	-1.316	[-0.068 - 0.016]	0.000	1358	0.209	0.620

Note: nominally significant results are italicized and FDR significant results are italicized and bolded

Supplementary Table S9 Associations between Cortical Thickness and SANS Avolition (Factor 3)

ROI	Partial *r*	SE	*t*-Score	95% CI	*I2*	*N*	*p-value*	FDR
Left Bankssts	-0.041	0.016	-1.306	[-0.107 - 0.026]	0.000	1266	0.213	0.601
Left Caudal Anterior Cingulate	0.030	0.015	0.965	[-0.036 - 0.095]	0.000	1362	0.351	0.681
Left Caudal Middle Frontal	-0.044	0.014	-1.560	[-0.104 - 0.016]	0.000	1351	0.141	0.506
Left Cuneus	0.008	0.015	0.263	[-0.057 - 0.074]	0.000	1275	0.796	0.967
Left Entorhinal	0.001	0.015	0.029	[-0.062 - 0.064]	0.000	1278	0.977	0.991
Left Fusiform	-0.048	0.015	-1.596	[-0.112 - 0.017]	0.000	1308	0.133	0.506
Left Inferior Parietal	0.046	0.016	1.457	[-0.022 - 0.113]	0.000	1234	0.167	0.532
Left Inferior Temporal	-0.028	0.019	-0.725	[-0.110 - 0.055]	0.000	1275	0.480	0.726
Left Isthmus Cingulate	0.003	0.012	0.123	[-0.050 - 0.056]	0.000	1361	0.904	0.991

ROI	Partial *r*	SE	*t*-Score	95% CI	*I2*	*N*	*p-value*	FDR
Left Lateral Occipital	-0.002	0.020	-0.052	[-0.087 - 0.083]	34.792	1296	0.960	0.991
Left Lateral Orbitofrontal	*-0.044*	*0.010*	*-2.165*	*[-0.087 - 0.000]*	*0.000*	*1364*	*0.048*	*0.374*
Left Lingual	-0.026	0.016	-0.795	[-0.096 - 0.044]	0.000	1339	0.440	0.712
Left Medial Orbitofrontal	-0.013	0.014	-0.489	[-0.071 - 0.045]	0.000	1347	0.633	0.896
Left Middle Temporal	-0.018	0.014	-0.643	[-0.077 - 0.042]	0.000	1218	0.530	0.784
Left Parahippocampal	0.005	0.018	0.129	[-0.074 - 0.083]	31.099	1342	0.899	0.991
Left Paracentral	-0.054	0.014	-1.943	[-0.113 - 0.006]	0.000	1360	0.072	0.409
Left Pars Opercularis	*-0.077*	*0.017*	*-2.213*	*[-0.150 - -0.002]*	*0.000*	*1323*	*0.044*	*0.374*
Left Pars Orbitalis	*-0.045*	*0.010*	*-2.285*	*[-0.088 - -0.003]*	*0.000*	*1327*	*0.038*	*0.374*
Left Pars Triangularis	***-0.086***	***0.010***	***-4.475***	***[-0.127 - -0.045]***	***0.000***	***1304***	***0.001***	***0.027***
Left Pericalcarine	0.002	0.020	0.053	[-0.083 - 0.088]	44.373	1350	0.958	0.991
Left Postcentral	-0.049	0.017	-1.440	[-0.123 - 0.024]	0.000	1336	0.172	0.532
Left Posterior Cingulate	-0.014	0.019	-0.359	[-0.097 - 0.069]	30.096	1363	0.725	0.954
Left Precentral	-0.063	0.016	-1.977	[-0.131 - 0.005]	0.000	1344	0.068	0.409
Left Precuneus	-0.018	0.011	-0.796	[-0.065 - 0.030]	0.000	1337	0.440	0.712
Left Rostral Anterior Cingulate	0.004	0.013	0.145	[-0.050 - 0.057]	0.000	1347	0.887	0.991
Left Rostral Middle Frontal	-0.042	0.011	-1.855	[-0.091 - 0.007]	0.000	1312	0.085	0.412
Left Superior Frontal	-0.060	0.014	-2.093	[-0.120 - 0.001]	9.684	1350	0.055	0.374
Left Superior Parietal	0.010	0.016	0.292	[-0.061 - 0.080]	0.000	1283	0.774	0.960
Left Superior Temporal	-0.041	0.016	-1.276	[-0.110 - 0.028]	0.000	1197	0.223	0.601
Left Supramarginal	-0.032	0.016	-0.986	[-0.100 - 0.037]	0.000	1216	0.341	0.681
Left Frontal Pole	-0.037	0.012	-1.573	[-0.087 - 0.013]	0.000	1368	0.138	0.506
Left Temporal Pole	*-0.066*	*0.011*	*-3.098*	*[-0.111 - -0.020]*	*0.000*	*1329*	*0.008*	*0.178*
Left Transverse Temporal	0.007	0.016	0.209	[-0.063 - 0.077]	0.000	1362	0.838	0.982
Left Insula	*-0.087*	*0.017*	*-2.518*	*[-0.160 - -0.013]*	*24.260*	*1365*	*0.025*	*0.334*
Right Bankssts	0.000	0.017	0.012	[-0.070 - 0.071]	0.000	1317	0.991	0.991
Right Caudal Anterior Cingulate	0.028	0.013	1.047	[-0.029 - 0.085]	0.000	1361	0.313	0.681
Right Caudal Middle Frontal	***-0.078***	***0.009***	***-4.258***	***[-0.116 - -0.039]***	***0.000***	***1337***	***0.001***	***0.027***
Right Cuneus	0.021	0.012	0.928	[-0.028 - 0.071]	0.000	1316	0.369	0.681
Right Entorhinal	-0.007	0.012	-0.290	[-0.057 - 0.043]	0.000	1164	0.776	0.960
Right Fusiform	-0.038	0.016	-1.207	[-0.104 - 0.029]	0.000	1281	0.247	0.601
Right Inferior Parietal	-0.004	0.020	-0.108	[-0.090 - 0.081]	0.000	1226	0.915	0.991
Right Inferior Temporal	-0.032	0.017	-0.932	[-0.107 - 0.042]	12.277	1283	0.367	0.681
Right Isthmus Cingulate	0.010	0.012	0.393	[-0.043 - 0.063]	0.000	1351	0.700	0.954
Right Lateral Occipital	-0.015	0.021	-0.353	[-0.106 - 0.076]	41.221	1287	0.729	0.954
Right Lateral Orbitofrontal	-0.038	0.012	-1.559	[-0.090 - 0.014]	0.000	1333	0.141	0.506
Right Lingual	-0.023	0.015	-0.773	[-0.085 - 0.040]	0.000	1350	0.452	0.715
Right Medial Orbitofrontal	-0.032	0.016	-1.002	[-0.100 - 0.036]	0.000	1316	0.333	0.681
Right Middle Temporal	-0.037	0.021	-0.893	[-0.125 - 0.052]	39.163	1255	0.387	0.681
Right Parahippocampal	0.005	0.013	0.210	[-0.050 - 0.061]	0.000	1322	0.837	0.982
Right Paracentral	-0.026	0.015	-0.886	[-0.090 - 0.037]	0.000	1361	0.390	0.681
Right Pars Opercularis	-0.048	0.018	-1.352	[-0.124 - 0.028]	9.199	1305	0.198	0.584
Right Pars Orbitalis	-0.033	0.013	-1.228	[-0.090 - 0.024]	0.000	1336	0.240	0.601
Right Pars Triangularis	-0.020	0.013	-0.731	[-0.077 - 0.038]	0.000	1305	0.477	0.726
Right Pericalcarine	-0.031	0.015	-0.995	[-0.097 - 0.036]	0.000	1350	0.337	0.681
Right Postcentral	-0.046	0.019	-1.214	[-0.127 - 0.035]	43.110	1342	0.245	0.601
Right Posterior Cingulate	-0.011	0.015	-0.368	[-0.074 - 0.052]	0.000	1364	0.718	0.954
Right Precentral	-0.034	0.010	-1.636	[-0.078 - 0.010]	0.000	1333	0.124	0.506
Right Precuneus	0.013	0.012	0.552	[-0.039 - 0.066]	0.000	1333	0.590	0.853
Right Rostral Anterior Cingulate	-0.022	0.013	-0.860	[-0.078 - 0.033]	0.000	1327	0.405	0.688
Right Rostral Middle Frontal	-0.043	0.011	-1.901	[-0.092 - 0.006]	0.000	1302	0.078	0.409
Right Superior Frontal	*-0.069*	*0.012*	*-2.953*	*[-0.118 - -0.019]*	*0.000*	*1341*	*0.010*	*0.178*

ROI	Partial *r*	SE	*t*-Score	95% CI	*I2*	*N*	*p-value*	FDR
Right Superior Parietal	0.009	0.014	0.333	[-0.051 - 0.069]	0.000	1314	0.744	0.955
Right Superior Temporal	-0.025	0.013	-0.923	[-0.082 - 0.033]	0.000	1234	0.372	0.681
Right Supramarginal	-0.064	0.022	-1.476	[-0.157 - 0.029]	43.570	1223	0.162	0.532
Right Frontal Pole	0.001	0.012	0.047	[-0.051 - 0.054]	0.000	1365	0.963	0.991
Right Temporal Pole	0.000	0.013	-0.016	[-0.056 - 0.055]	0.000	1225	0.987	0.991
Right Transverse Temporal	-0.032	0.014	-1.157	[-0.090 - 0.027]	0.000	1363	0.267	0.626
Right Insula	-0.074	0.017	-2.142	[-0.148 - 0.000]	0.000	1363	0.050	0.374
Left Thickness	-0.042	0.015	-1.434	[-0.104 - 0.021]	0.000	1370	0.174	1.000
Right Thickness	-0.040	0.015	-1.316	[-0.104 - 0.025]	0.000	1370	0.209	1.000
Left Surface Area	*-0.090*	*0.013*	*-3.320*	*[-0.147 - -0.032]*	*0.000*	*1370*	*0.005*	*1.000*
Right Surface Area	*-0.090*	*0.012*	*-3.725*	*[-0.141 - -0.038]*	*0.000*	*1370*	*0.002*	*1.000*
Mean Bankssts	-0.026	0.016	-0.794	[-0.097 - 0.044]	0.000	1230	0.440	0.748
Mean Caudal Anterior Cingulate	0.038	0.017	1.141	[-0.033 - 0.109]	0.000	1354	0.273	0.580
Mean Caudal Middle Frontal	*-0.066*	*0.011*	*-2.897*	*[-0.115 - -0.017]*	*0.000*	*1323*	*0.012*	*0.199*
Mean Cuneus	0.020	0.014	0.712	[-0.041 - 0.081]	0.000	1243	0.488	0.759
Mean Entorhinal	0.001	0.014	0.029	[-0.059 - 0.060]	0.000	1137	0.977	0.977
Mean Fusiform	-0.044	0.017	-1.280	[-0.116 - 0.029]	11.202	1240	0.221	0.537
Mean Inferior Parietal	0.021	0.018	0.566	[-0.058 - 0.099]	0.000	1160	0.580	0.822
Mean Inferior Temporal	-0.042	0.023	-0.892	[-0.142 - 0.059]	60.693	1227	0.387	0.693
Mean Isthmus Cingulate	0.006	0.013	0.245	[-0.049 - 0.062]	0.000	1345	0.810	0.927
Mean Lateral Occipital	-0.005	0.022	-0.110	[-0.101 - 0.091]	44.717	1237	0.914	0.971
Mean Lateral Orbitofrontal	-0.043	0.011	-1.970	[-0.090 - 0.004]	0.000	1327	0.069	0.327
Mean Lingual	-0.032	0.016	-0.989	[-0.101 - 0.038]	0.000	1328	0.339	0.641
Mean Medial Orbitofrontal	-0.022	0.016	-0.707	[-0.089 - 0.045]	0.000	1298	0.491	0.759
Mean Middle Temporal	-0.026	0.019	-0.656	[-0.109 - 0.058]	24.911	1162	0.523	0.772
Mean Parahippocampal	0.008	0.016	0.235	[-0.063 - 0.078]	3.696	1305	0.818	0.927
Mean Paracentral	-0.038	0.015	-1.292	[-0.100 - 0.025]	0.000	1352	0.217	0.537
Mean Pars Opercularis	-0.073	0.020	-1.844	[-0.157 - 0.012]	21.844	1273	0.086	0.327
Mean Pars Orbitalis	*-0.056*	*0.009*	*-3.233*	*[-0.093 - -0.019]*	*0.000*	*1301*	*0.006*	*0.199*
Mean Pars Triangularis	*-0.058*	*0.013*	*-2.315*	*[-0.112 - -0.004]*	*0.000*	*1256*	*0.036*	*0.213*
Mean Pericalcarine	-0.012	0.017	-0.346	[-0.086 - 0.062]	0.000	1340	0.735	0.892
Mean Postcentral	-0.060	0.017	-1.755	[-0.132 - 0.013]	0.000	1316	0.101	0.344
Mean Posterior Cingulate	-0.014	0.017	-0.405	[-0.088 - 0.060]	18.157	1358	0.692	0.892
Mean Precentral	*-0.060*	*0.013*	*-2.297*	*[-0.116 - -0.004]*	*0.000*	*1318*	*0.038*	*0.213*
Mean Precuneus	-0.001	0.012	-0.059	[-0.051 - 0.049]	0.000	1311	0.954	0.977
Mean Rostral Anterior Cingulate	-0.005	0.013	-0.175	[-0.061 - 0.052]	0.000	1309	0.864	0.948
Mean Rostral Middle Frontal	-0.044	0.012	-1.886	[-0.095 - 0.006]	0.000	1263	0.080	0.327
Mean Superior Frontal	*-0.066*	*0.012*	*-2.690*	*[-0.119 - -0.014]*	*0.000*	*1325*	*0.018*	*0.199*
Mean Superior Parietal	0.012	0.016	0.375	[-0.056 - 0.080]	0.000	1248	0.713	0.892
Mean Superior Temporal	-0.036	0.015	-1.233	[-0.099 - 0.027]	0.000	1132	0.238	0.539
Mean Supramarginal	-0.053	0.018	-1.468	[-0.131 - 0.025]	0.000	1147	0.164	0.507
Mean Frontal Pole	-0.020	0.010	-1.001	[-0.062 - 0.023]	0.000	1363	0.334	0.641
Mean Temporal Pole	-0.030	0.012	-1.288	[-0.081 - 0.020]	0.000	1206	0.219	0.537
Mean Transverse Temporal	-0.015	0.015	-0.487	[-0.079 - 0.050]	0.000	1356	0.634	0.862
Mean Insula	*-0.081*	*0.017*	*-2.322*	*[-0.154 - -0.006]*	*0.000*	*1358*	*0.036*	*0.213*

Note: nominally significant results are italicized and FDR significant results are italicized and bolded

Supplementary Table S10 Associations between Cortical Thickness and SANS EXP

ROI	Partial *r*	SE	*t*-Score	95% CI	*I2*	*N*	*p-value*	FDR
Left Bankssts	-0.046	0.014	-1.594	[-0.108 - 0.016]	0.000	1266	0.133	0.414
Left Caudal Anterior Cingulate	0.050	0.013	1.966	[-0.005 - 0.104]	0.000	1362	0.069	0.337
Left Caudal Middle Frontal	-0.005	0.014	-0.184	[-0.066 - 0.056]	0.000	1351	0.857	0.893
Left Cuneus	-0.004	0.018	-0.125	[-0.080 - 0.071]	0.000	1275	0.902	0.916
Left Entorhinal	0.007	0.009	0.423	[-0.030 - 0.044]	0.000	1278	0.679	0.839
Left Fusiform	-0.058	0.014	-2.073	[-0.118 - 0.002]	0.000	1308	0.057	0.298
Left Inferior Parietal	-0.011	0.016	-0.353	[-0.080 - 0.057]	0.000	1234	0.729	0.867
Left Inferior Temporal	-0.007	0.012	-0.317	[-0.057 - 0.042]	0.000	1275	0.756	0.867
Left Isthmus Cingulate	-0.029	0.013	-1.128	[-0.084 - 0.026]	0.000	1361	0.278	0.601
Left Lateral Occipital	-0.043	0.015	-1.467	[-0.106 - 0.020]	0.000	1296	0.165	0.426
Left Lateral Orbitofrontal	-0.032	0.014	-1.118	[-0.093 - 0.029]	0.000	1364	0.282	0.601
Left Lingual	-0.047	0.016	-1.449	[-0.115 - 0.022]	0.000	1339	0.169	0.426
Left Medial Orbitofrontal	-0.051	0.015	-1.722	[-0.114 - 0.013]	0.000	1347	0.107	0.405
Left Middle Temporal	-0.026	0.012	-1.095	[-0.076 - 0.025]	0.000	1218	0.292	0.601
Left Parahippocampal	-0.023	0.015	-0.772	[-0.088 - 0.042]	0.000	1342	0.453	0.671
Left Paracentral	-0.009	0.016	-0.301	[-0.077 - 0.058]	0.000	1360	0.768	0.867
Left Pars Opercularis	*-0.069*	*0.013*	*-2.746*	*[-0.123 - -0.015]*	*0.000*	*1323*	*0.016*	*0.268*
Left Pars Orbitalis	*-0.074*	*0.009*	*-4.115*	*[-0.113 - -0.036]*	*0.000*	*1327*	*0.001*	*0.071*
Left Pars Triangularis	*-0.055*	*0.013*	*-2.181*	*[-0.109 - -0.001]*	*0.000*	*1304*	*0.047*	*0.298*
Left Pericalcarine	0.006	0.016	0.181	[-0.061 - 0.073]	0.000	1350	0.859	0.893
Left Postcentral	-0.040	0.014	-1.465	[-0.098 - 0.018]	0.000	1336	0.165	0.426
Left Posterior Cingulate	-0.058	0.015	-1.917	[-0.121 - 0.007]	0.000	1363	0.076	0.344
Left Precentral	-0.055	0.015	-1.779	[-0.120 - 0.011]	0.000	1344	0.097	0.389
Left Precuneus	-0.032	0.015	-1.054	[-0.097 - 0.033]	0.000	1337	0.310	0.601
Left Rostral Anterior Cingulate	0.046	0.013	1.778	[-0.009 - 0.101]	0.000	1347	0.097	0.389
Left Rostral Middle Frontal	*-0.058*	*0.013*	*-2.298*	*[-0.111 - -0.004]*	*0.000*	*1312*	*0.038*	*0.298*
Left Superior Frontal	*-0.081*	*0.011*	*-3.530*	*[-0.130 - -0.032]*	*0.000*	*1350*	*0.003*	*0.075*
Left Superior Parietal	-0.014	0.021	-0.327	[-0.106 - 0.078]	49.127	1283	0.748	0.867
Left Superior Temporal	*-0.060*	*0.012*	*-2.492*	*[-0.111 - -0.008]*	*0.000*	*1197*	*0.026*	*0.298*
Left Supramarginal	*-0.062*	*0.014*	*-2.249*	*[-0.122 - -0.003]*	*0.000*	*1216*	*0.041*	*0.298*
Left Frontal Pole	-0.014	0.013	-0.551	[-0.068 - 0.040]	0.000	1368	0.590	0.787
Left Temporal Pole	0.008	0.009	0.449	[-0.031 - 0.047]	0.000	1329	0.661	0.832
Left Transverse Temporal	-0.008	0.012	-0.339	[-0.061 - 0.044]	0.000	1362	0.740	0.867
Left Insula	-0.026	0.016	-0.820	[-0.092 - 0.041]	0.000	1365	0.426	0.671
Right Bankssts	-0.059	0.026	-1.118	[-0.171 - 0.054]	70.770	1317	0.282	0.601
Right Caudal Anterior Cingulate	-0.003	0.020	-0.067	[-0.087 - 0.082]	0.000	1361	0.948	0.948
Right Caudal Middle Frontal	*-0.066*	*0.015*	*-2.180*	*[-0.131 - -0.001]*	*0.000*	*1337*	*0.047*	*0.298*
Right Cuneus	-0.053	0.018	-1.477	[-0.130 - 0.024]	15.034	1316	0.162	0.426
Right Entorhinal	0.034	0.016	1.067	[-0.034 - 0.101]	0.000	1164	0.304	0.601
Right Fusiform	-0.016	0.013	-0.578	[-0.073 - 0.042]	0.000	1281	0.572	0.783
Right Inferior Parietal	-0.033	0.020	-0.827	[-0.118 - 0.053]	0.000	1226	0.422	0.671
Right Inferior Temporal	-0.021	0.013	-0.807	[-0.077 - 0.035]	0.000	1283	0.433	0.671
Right Isthmus Cingulate	-0.012	0.013	-0.454	[-0.069 - 0.045]	0.000	1351	0.657	0.832
Right Lateral Occipital	-0.024	0.015	-0.788	[-0.091 - 0.042]	0.000	1287	0.444	0.671
Right Lateral Orbitofrontal	-0.048	0.016	-1.518	[-0.114 - 0.020]	0.000	1333	0.151	0.426
Right Lingual	-0.036	0.013	-1.397	[-0.091 - 0.019]	0.000	1350	0.184	0.447
Right Medial Orbitofrontal	-0.048	0.014	-1.642	[-0.109 - 0.015]	0.000	1316	0.123	0.414
Right Middle Temporal	-0.006	0.018	-0.185	[-0.082 - 0.069]	0.000	1255	0.856	0.893
Right Parahippocampal	0.011	0.010	0.575	[-0.031 - 0.054]	0.000	1322	0.574	0.783
Right Paracentral	-0.030	0.016	-0.969	[-0.098 - 0.037]	0.000	1361	0.349	0.642
Right Pars Opercularis	-0.055	0.013	-2.113	[-0.110 - 0.001]	0.000	1305	0.053	0.298
Right Pars Orbitalis	-0.015	0.013	-0.573	[-0.072 - 0.042]	0.000	1336	0.576	0.783

ROI	Partial *r*	SE	*t*-Score	95% CI	*I2*	*N*	*p-value*	FDR
Right Pars Triangularis	*-0.058*	*0.012*	*-2.457*	*[-0.108 - -0.007]*	*0.000*	*1305*	*0.028*	*0.298*
Right Pericalcarine	-0.024	0.015	-0.795	[-0.087 - 0.040]	0.000	1350	0.440	0.671
Right Postcentral	-0.020	0.012	-0.855	[-0.070 - 0.030]	0.000	1342	0.407	0.671
Right Posterior Cingulate	-0.015	0.017	-0.456	[-0.086 - 0.056]	0.000	1364	0.655	0.832
Right Precentral	-0.038	0.012	-1.591	[-0.088 - 0.013]	0.000	1333	0.134	0.414
Right Precuneus	-0.007	0.018	-0.195	[-0.082 - 0.068]	0.000	1333	0.848	0.893
Right Rostral Anterior Cingulate	0.006	0.017	0.171	[-0.066 - 0.078]	0.000	1327	0.867	0.893
Right Rostral Middle Frontal	-0.057	0.014	-2.077	[-0.115 - 0.002]	0.000	1302	0.057	0.298
Right Superior Frontal	*-0.081*	*0.011*	*-3.593*	*[-0.129 - -0.033]*	*0.000*	*1341*	*0.003*	*0.075*
Right Superior Parietal	-0.010	0.018	-0.287	[-0.086 - 0.065]	22.714	1314	0.778	0.867
Right Superior Temporal	-0.040	0.020	-1.014	[-0.124 - 0.045]	0.000	1234	0.328	0.619
Right Supramarginal	-0.051	0.016	-1.645	[-0.117 - 0.016]	0.000	1223	0.122	0.414
Right Frontal Pole	-0.019	0.010	-0.916	[-0.063 - 0.025]	0.000	1365	0.375	0.671
Right Temporal Pole	-0.009	0.006	-0.732	[-0.037 - 0.018]	0.000	1225	0.476	0.689
Right Transverse Temporal	-0.018	0.011	-0.770	[-0.067 - 0.032]	0.000	1363	0.454	0.671
Right Insula	-0.034	0.016	-1.074	[-0.100 - 0.033]	0.000	1363	0.301	0.601
Left Thickness	-0.049	0.013	-1.847	[-0.105 - 0.008]	0.000	1370	0.086	1.000
Right Thickness	-0.052	0.013	-2.044	[-0.106 - 0.003]	0.000	1370	0.060	1.000
Left Surface Area	-0.022	0.014	-0.754	[-0.084 - 0.040]	0.000	1370	0.463	1.000
Right Surface Area	-0.014	0.015	-0.454	[-0.078 - 0.051]	0.000	1370	0.657	1.000
Mean Bankssts	-0.071	0.024	-1.475	[-0.173 - 0.032]	63.345	1230	0.162	0.460
Mean Caudal Anterior Cingulate	0.035	0.016	1.075	[-0.035 - 0.104]	0.000	1354	0.300	0.565
Mean Caudal Middle Frontal	-0.036	0.015	-1.199	[-0.101 - 0.029]	0.000	1323	0.250	0.516
Mean Cuneus	-0.042	0.021	-1.004	[-0.132 - 0.048]	47.918	1243	0.332	0.565
Mean Entorhinal	0.015	0.011	0.719	[-0.030 - 0.061]	0.000	1137	0.484	0.629
Mean Fusiform	-0.046	0.013	-1.802	[-0.101 - 0.009]	0.000	1240	0.093	0.316
Mean Inferior Parietal	-0.033	0.018	-0.888	[-0.111 - 0.046]	0.000	1160	0.390	0.576
Mean Inferior Temporal	-0.009	0.012	-0.409	[-0.059 - 0.040]	0.000	1227	0.689	0.744
Mean Isthmus Cingulate	-0.023	0.013	-0.895	[-0.078 - 0.032]	0.000	1345	0.386	0.576
Mean Lateral Occipital	-0.039	0.015	-1.272	[-0.104 - 0.027]	0.000	1237	0.224	0.508
Mean Lateral Orbitofrontal	-0.039	0.015	-1.310	[-0.102 - 0.025]	0.000	1327	0.211	0.508
Mean Lingual	-0.051	0.015	-1.748	[-0.113 - 0.012]	0.000	1328	0.102	0.316
Mean Medial Orbitofrontal	-0.056	0.015	-1.885	[-0.120 - 0.008]	0.000	1298	0.080	0.304
Mean Middle Temporal	-0.023	0.016	-0.742	[-0.090 - 0.044]	0.000	1162	0.470	0.629
Mean Parahippocampal	-0.007	0.013	-0.278	[-0.064 - 0.049]	0.000	1305	0.785	0.785
Mean Paracentral	-0.021	0.017	-0.641	[-0.092 - 0.050]	0.000	1352	0.532	0.646
Mean Pars Opercularis	*-0.072*	*0.013*	*-2.756*	*[-0.128 - -0.016]*	*0.000*	*1273*	*0.015*	*0.131*
Mean Pars Orbitalis	*-0.062*	*0.011*	*-2.911*	*[-0.108 - -0.016]*	*0.000*	*1301*	*0.011*	*0.129*
Mean Pars Triangularis	*-0.075*	*0.012*	*-2.974*	*[-0.128 - -0.021]*	*0.000*	*1256*	*0.010*	*0.129*
Mean Pericalcarine	-0.011	0.015	-0.373	[-0.073 - 0.052]	0.000	1340	0.715	0.744
Mean Postcentral	-0.034	0.012	-1.397	[-0.087 - 0.018]	0.000	1316	0.184	0.482
Mean Posterior Cingulate	-0.038	0.016	-1.180	[-0.108 - 0.031]	0.000	1358	0.258	0.516
Mean Precentral	*-0.059*	*0.013*	*-2.275*	*[-0.115 - -0.003]*	*0.000*	*1318*	*0.039*	*0.190*
Mean Precuneus	-0.020	0.018	-0.556	[-0.096 - 0.056]	0.000	1311	0.587	0.688
Mean Rostral Anterior Cingulate	0.030	0.016	0.946	[-0.038 - 0.097]	0.000	1309	0.360	0.576
Mean Rostral Middle Frontal	*-0.067*	*0.013*	*-2.498*	*[-0.123 - -0.009]*	*0.000*	*1263*	*0.026*	*0.166*
Mean Superior Frontal	*-0.083*	*0.012*	*-3.590*	*[-0.132 - -0.033]*	*0.000*	*1325*	*0.003*	*0.100*
Mean Superior Parietal	-0.019	0.021	-0.446	[-0.110 - 0.073]	46.494	1248	0.663	0.744
Mean Superior Temporal	-0.059	0.015	-1.973	[-0.122 - 0.005]	0.000	1132	0.069	0.291
Mean Supramarginal	*-0.070*	*0.014*	*-2.427*	*[-0.131 - -0.008]*	*0.000*	*1147*	*0.029*	*0.166*
Mean Frontal Pole	-0.015	0.010	-0.694	[-0.059 - 0.030]	0.000	1363	0.499	0.629

ROI	Partial *r*	SE	*t*-Score	95% CI	*I2*	*N*	*p-value*	FDR
Mean Temporal Pole	0.004	0.006	0.363	[-0.022 - 0.031]	0.000	1206	0.722	0.744
Mean Transverse Temporal	-0.016	0.011	-0.740	[-0.062 - 0.030]	0.000	1356	0.471	0.629
Mean Insula	-0.032	0.016	-1.024	[-0.099 - 0.035]	0.000	1358	0.323	0.565

Note: nominally significant results are italicized and FDR significant results are italicized and bolded

Supplementary Table S11 Associations between Cortical Thickness and SANS Blunted Affect (Factor 4)

ROI	Partial *r*	SE	*t*-Score	95% CI	*I2*	*N*	*p-value*	FDR
Left Bankssts	-0.041	0.014	-1.447	[-0.102 - 0.020]	0.000	1266	0.170	0.578
Left Caudal Anterior Cingulate	*0.051*	*0.011*	*2.296*	*[0.003 - 0.098]*	*0.000*	*1362*	*0.038*	*0.458*
Left Caudal Middle Frontal	0.011	0.014	0.414	[-0.047 - 0.069]	0.000	1351	0.685	0.867
Left Cuneus	-0.009	0.020	-0.231	[-0.096 - 0.077]	0.000	1275	0.820	0.950
Left Entorhinal	0.009	0.008	0.584	[-0.024 - 0.041]	0.000	1278	0.568	0.867
Left Fusiform	-0.045	0.014	-1.668	[-0.104 - 0.013]	0.000	1308	0.118	0.458
Left Inferior Parietal	-0.001	0.017	-0.023	[-0.072 - 0.070]	0.000	1234	0.982	0.982
Left Inferior Temporal	0.001	0.012	0.025	[-0.052 - 0.053]	0.000	1275	0.980	0.982
Left Isthmus Cingulate	-0.028	0.014	-1.037	[-0.087 - 0.030]	0.000	1361	0.317	0.775
Left Lateral Occipital	-0.037	0.016	-1.131	[-0.107 - 0.033]	0.000	1296	0.277	0.754
Left Lateral Orbitofrontal	-0.028	0.014	-0.971	[-0.090 - 0.034]	0.000	1364	0.348	0.778
Left Lingual	-0.038	0.018	-1.051	[-0.115 - 0.040]	0.000	1339	0.311	0.775
Left Medial Orbitofrontal	-0.054	0.015	-1.832	[-0.117 - 0.009]	0.000	1347	0.088	0.458
Left Middle Temporal	-0.013	0.011	-0.551	[-0.062 - 0.036]	0.000	1218	0.591	0.867
Left Parahippocampal	-0.015	0.016	-0.450	[-0.085 - 0.056]	13.620	1342	0.660	0.867
Left Paracentral	0.006	0.015	0.202	[-0.058 - 0.070]	0.000	1360	0.843	0.955
Left Pars Opercularis	*-0.060*	*0.013*	*-2.265*	*[-0.117 - -0.003]*	*0.000*	*1323*	*0.040*	*0.458*
Left Pars Orbitalis	*-0.081*	*0.010*	*-3.987*	*[-0.125 - -0.038]*	*0.000*	*1327*	*0.001*	*0.092*
Left Pars Triangularis	-0.044	0.012	-1.789	[-0.097 - 0.009]	0.000	1304	0.095	0.458
Left Pericalcarine	0.004	0.017	0.119	[-0.067 - 0.075]	0.000	1350	0.907	0.976
Left Postcentral	-0.031	0.014	-1.142	[-0.090 - 0.028]	0.000	1336	0.273	0.754
Left Posterior Cingulate	-0.047	0.014	-1.650	[-0.108 - 0.014]	0.000	1363	0.121	0.458
Left Precentral	-0.040	0.015	-1.365	[-0.102 - 0.023]	0.000	1344	0.194	0.601
Left Precuneus	-0.023	0.015	-0.752	[-0.088 - 0.043]	0.000	1337	0.464	0.867
Left Rostral Anterior Cingulate	*0.052*	*0.011*	*2.240*	*[0.002 - 0.101]*	*0.000*	*1347*	*0.042*	*0.458*
Left Rostral Middle Frontal	-0.047	0.014	-1.731	[-0.106 - 0.011]	0.000	1312	0.105	0.458
Left Superior Frontal	*-0.067*	*0.011*	*-3.088*	*[-0.114 - -0.021]*	*0.000*	*1350*	*0.008*	*0.182*
Left Superior Parietal	-0.004	0.021	-0.086	[-0.093 - 0.086]	45.814	1283	0.933	0.976
Left Superior Temporal	-0.041	0.011	-1.822	[-0.089 - 0.007]	0.000	1197	0.090	0.458
Left Supramarginal	-0.052	0.014	-1.877	[-0.112 - 0.007]	0.000	1216	0.082	0.458
Left Frontal Pole	-0.008	0.013	-0.288	[-0.064 - 0.049]	0.000	1368	0.777	0.927
Left Temporal Pole	0.011	0.011	0.509	[-0.036 - 0.058]	0.000	1329	0.619	0.867
Left Transverse Temporal	0.009	0.011	0.420	[-0.037 - 0.054]	0.000	1362	0.681	0.867
Left Insula	-0.020	0.015	-0.652	[-0.086 - 0.046]	4.239	1365	0.525	0.867
Right Bankssts	-0.049	0.026	-0.958	[-0.159 - 0.061]	70.254	1317	0.355	0.778
Right Caudal Anterior Cingulate	-0.004	0.018	-0.120	[-0.082 - 0.073]	0.000	1361	0.906	0.976
Right Caudal Middle Frontal	-0.050	0.014	-1.730	[-0.111 - 0.012]	0.000	1337	0.106	0.458
Right Cuneus	-0.060	0.022	-1.363	[-0.154 - 0.035]	56.887	1316	0.194	0.601
Right Entorhinal	0.033	0.016	1.033	[-0.036 - 0.102]	0.000	1164	0.319	0.775
Right Fusiform	-0.008	0.013	-0.311	[-0.066 - 0.049]	0.000	1281	0.760	0.923
Right Inferior Parietal	-0.026	0.020	-0.650	[-0.113 - 0.061]	0.000	1226	0.526	0.867
Right Inferior Temporal	-0.013	0.013	-0.508	[-0.068 - 0.042]	0.000	1283	0.619	0.867
Right Isthmus Cingulate	-0.015	0.015	-0.482	[-0.080 - 0.051]	0.000	1351	0.637	0.867
Right Lateral Occipital	-0.015	0.016	-0.461	[-0.086 - 0.055]	0.000	1287	0.652	0.867

ROI	Partial *r*	SE	*t*-Score	95% CI	*I2*	*N*	*p-value*	FDR
Right Lateral Orbitofrontal	-0.047	0.016	-1.525	[-0.114 - 0.019]	0.000	1333	0.150	0.535
Right Lingual	-0.022	0.014	-0.785	[-0.082 - 0.038]	0.000	1350	0.445	0.867
Right Medial Orbitofrontal	-0.049	0.014	-1.690	[-0.110 - 0.013]	0.000	1316	0.113	0.458
Right Middle Temporal	0.001	0.019	0.037	[-0.078 - 0.081]	0.000	1255	0.971	0.982
Right Parahippocampal	0.019	0.011	0.840	[-0.030 - 0.068]	0.000	1322	0.415	0.867
Right Paracentral	-0.018	0.016	-0.547	[-0.087 - 0.052]	0.000	1361	0.593	0.867
Right Pars Opercularis	-0.044	0.012	-1.768	[-0.096 - 0.009]	0.000	1305	0.099	0.458
Right Pars Orbitalis	-0.010	0.013	-0.382	[-0.067 - 0.047]	0.000	1336	0.708	0.876
Right Pars Triangularis	-0.054	0.013	-2.128	[-0.108 - 0.000]	0.000	1305	0.052	0.458
Right Pericalcarine	-0.013	0.016	-0.410	[-0.079 - 0.054]	0.000	1350	0.688	0.867
Right Postcentral	-0.013	0.012	-0.521	[-0.064 - 0.039]	0.000	1342	0.610	0.867
Right Posterior Cingulate	-0.007	0.016	-0.227	[-0.078 - 0.063]	0.000	1364	0.824	0.950
Right Precentral	-0.026	0.013	-0.997	[-0.080 - 0.029]	0.000	1333	0.336	0.778
Right Precuneus	0.005	0.018	0.137	[-0.073 - 0.083]	0.000	1333	0.893	0.976
Right Rostral Anterior Cingulate	0.016	0.015	0.543	[-0.047 - 0.079]	0.000	1327	0.596	0.867
Right Rostral Middle Frontal	-0.057	0.014	-1.965	[-0.118 - 0.005]	0.000	1302	0.070	0.458
Right Superior Frontal	*-0.069*	*0.011*	*-3.197*	*[-0.115 - -0.023]*	*0.000*	*1341*	*0.006*	*0.182*
Right Superior Parietal	-0.004	0.018	-0.097	[-0.083 - 0.076]	30.079	1314	0.924	0.976
Right Superior Temporal	-0.024	0.019	-0.628	[-0.106 - 0.058]	0.000	1234	0.540	0.867
Right Supramarginal	-0.038	0.015	-1.268	[-0.103 - 0.027]	0.000	1223	0.225	0.666
Right Frontal Pole	-0.015	0.010	-0.710	[-0.059 - 0.029]	0.000	1365	0.489	0.867
Right Temporal Pole	-0.007	0.007	-0.439	[-0.038 - 0.025]	0.000	1225	0.667	0.867
Right Transverse Temporal	-0.010	0.010	-0.461	[-0.054 - 0.035]	0.000	1363	0.652	0.867
Right Insula	-0.021	0.016	-0.639	[-0.089 - 0.048]	0.000	1363	0.533	0.867
Left Thickness	-0.035	0.013	-1.330	[-0.092 - 0.022]	0.000	1370	0.205	1.000
Right Thickness	-0.038	0.014	-1.396	[-0.096 - 0.020]	0.000	1370	0.184	1.000
Left Surface Area	-0.009	0.013	-0.336	[-0.066 - 0.048]	0.000	1370	0.742	1.000
Right Surface Area	-0.003	0.014	-0.106	[-0.063 - 0.057]	0.000	1370	0.917	1.000
Mean Bankssts	-0.042	0.020	-1.058	[-0.126 - 0.043]	0.000	1230	0.308	0.633
Mean Caudal Anterior Cingulate	0.036	0.014	1.287	[-0.024 - 0.095]	0.000	1354	0.219	0.620
Mean Caudal Middle Frontal	-0.019	0.014	-0.665	[-0.079 - 0.042]	0.000	1323	0.517	0.703
Mean Cuneus	-0.053	0.025	-1.039	[-0.160 - 0.056]	66.200	1243	0.316	0.633
Mean Entorhinal	0.017	0.010	0.876	[-0.025 - 0.059]	0.000	1137	0.396	0.651
Mean Fusiform	-0.036	0.012	-1.465	[-0.089 - 0.017]	0.000	1240	0.165	0.564
Mean Inferior Parietal	-0.025	0.018	-0.673	[-0.102 - 0.054]	0.000	1160	0.512	0.703
Mean Inferior Temporal	-0.002	0.012	-0.101	[-0.055 - 0.050]	0.000	1227	0.921	0.921
Mean Isthmus Cingulate	-0.023	0.014	-0.813	[-0.085 - 0.038]	0.000	1345	0.430	0.664
Mean Lateral Occipital	-0.031	0.017	-0.938	[-0.102 - 0.040]	0.000	1237	0.364	0.651
Mean Lateral Orbitofrontal	-0.036	0.015	-1.220	[-0.099 - 0.027]	0.000	1327	0.243	0.633
Mean Lingual	-0.038	0.016	-1.172	[-0.107 - 0.032]	0.000	1328	0.261	0.633
Mean Medial Orbitofrontal	-0.060	0.015	-2.050	[-0.123 - 0.003]	0.000	1298	0.060	0.289
Mean Middle Temporal	-0.014	0.016	-0.425	[-0.082 - 0.055]	0.000	1162	0.677	0.874
Mean Parahippocampal	0.004	0.014	0.148	[-0.057 - 0.065]	0.000	1305	0.885	0.921
Mean Paracentral	-0.006	0.016	-0.187	[-0.075 - 0.063]	0.000	1352	0.854	0.921
Mean Pars Opercularis	*-0.060*	*0.013*	*-2.309*	*[-0.116 - -0.004]*	*0.000*	*1273*	*0.037*	*0.289*
Mean Pars Orbitalis	*-0.063*	*0.012*	*-2.637*	*[-0.115 - -0.012]*	*0.000*	*1301*	*0.020*	*0.289*
Mean Pars Triangularis	*-0.065*	*0.013*	*-2.493*	*[-0.121 - -0.009]*	*0.000*	*1256*	*0.026*	*0.289*
Mean Pericalcarine	-0.005	0.015	-0.175	[-0.072 - 0.061]	0.000	1340	0.864	0.921
Mean Postcentral	-0.027	0.013	-1.062	[-0.081 - 0.027]	0.000	1316	0.306	0.633
Mean Posterior Cingulate	-0.028	0.016	-0.883	[-0.097 - 0.041]	0.000	1358	0.392	0.651
Mean Precentral	-0.044	0.013	-1.651	[-0.101 - 0.013]	0.000	1318	0.121	0.514

ROI	Partial *r*	SE	*t*-Score	95% CI	*I2*	*N*	*p-value*	FDR
Mean Precuneus	-0.036	0.021	-0.864	[-0.126 - 0.054]	49.730	1311	0.402	0.651
Mean Rostral Anterior Cingulate	0.039	0.013	1.462	[-0.018 - 0.095]	0.000	1309	0.166	0.564
Mean Rostral Middle Frontal	*-0.062*	*0.014*	*-2.176*	*[-0.123 - -0.001]*	*0.000*	*1263*	*0.047*	*0.289*
Mean Superior Frontal	*-0.070*	*0.011*	*-3.198*	*[-0.117 - -0.023]*	*0.000*	*1325*	*0.006*	*0.219*
Mean Superior Parietal	-0.009	0.021	-0.219	[-0.099 - 0.081]	43.663	1248	0.830	0.921
Mean Superior Temporal	-0.038	0.014	-1.331	[-0.098 - 0.023]	0.000	1132	0.205	0.620
Mean Supramarginal	-0.058	0.014	-2.100	[-0.117 - 0.001]	0.000	1147	0.054	0.289
Mean Frontal Pole	-0.008	0.011	-0.366	[-0.054 - 0.038]	0.000	1363	0.720	0.874
Mean Temporal Pole	0.006	0.009	0.371	[-0.031 - 0.043]	0.000	1206	0.716	0.874
Mean Transverse Temporal	-0.002	0.009	-0.130	[-0.042 - 0.038]	0.000	1356	0.899	0.921
Mean Insula	-0.021	0.016	-0.669	[-0.088 - 0.046]	0.000	1358	0.515	0.703

Note: nominally significant results are italicized and FDR significant results are italicized and bolded

Supplementary Table S12 Associations between Cortical Thickness and SANS Alogia (Factor 5)

ROI	Partial *r*	SE	*t*-Score	95% CI	*I2*	*N*	*p-value*	FDR
Left Bankssts	-0.044	0.015	-1.452	[-0.109 - 0.021]	0.000	1266	0.168	0.327
Left Caudal Anterior Cingulate	0.035	0.014	1.240	[-0.025 - 0.094]	0.000	1362	0.235	0.370
Left Caudal Middle Frontal	*-0.058*	*0.011*	*-2.550*	*[-0.107 - -0.009]*	*0.000*	*1351*	*0.023*	*0.117*
Left Cuneus	0.003	0.016	0.092	[-0.066 - 0.072]	0.000	1275	0.928	0.954
Left Entorhinal	-0.006	0.017	-0.176	[-0.079 - 0.067]	22.784	1278	0.863	0.902
Left Fusiform	*-0.078*	*0.016*	*-2.402*	*[-0.147 - -0.008]*	*0.000*	*1308*	*0.031*	*0.117*
Left Inferior Parietal	-0.042	0.015	-1.380	[-0.107 - 0.023]	0.000	1234	0.189	0.348
Left Inferior Temporal	-0.033	0.013	-1.238	[-0.089 - 0.024]	0.000	1275	0.236	0.370
Left Isthmus Cingulate	-0.024	0.011	-1.126	[-0.069 - 0.021]	0.000	1361	0.279	0.383
Left Lateral Occipital	*-0.053*	*0.011*	*-2.395*	*[-0.101 - -0.006]*	*0.000*	*1296*	*0.031*	*0.117*
Left Lateral Orbitofrontal	-0.036	0.015	-1.177	[-0.101 - 0.030]	0.000	1364	0.259	0.382
Left Lingual	*-0.068*	*0.011*	*-2.991*	*[-0.117 - -0.019]*	*0.000*	*1339*	*0.010*	*0.110*
Left Medial Orbitofrontal	-0.024	0.013	-0.902	[-0.082 - 0.033]	0.000	1347	0.382	0.464
Left Middle Temporal	-0.060	0.014	-2.144	[-0.119 - 0.000]	0.000	1218	0.050	0.151
Left Parahippocampal	-0.056	0.014	-1.981	[-0.116 - 0.005]	0.000	1342	0.068	0.185
Left Paracentral	-0.055	0.014	-1.948	[-0.116 - 0.006]	0.000	1360	0.072	0.188
Left Pars Opercularis	*-0.082*	*0.016*	*-2.497*	*[-0.151 - -0.012]*	*0.000*	*1323*	*0.026*	*0.117*
Left Pars Orbitalis	-0.026	0.012	-1.122	[-0.076 - 0.024]	0.000	1327	0.281	0.383
Left Pars Triangularis	*-0.079*	*0.017*	*-2.368*	*[-0.149 - -0.007]*	*0.000*	*1304*	*0.033*	*0.117*
Left Pericalcarine	0.002	0.017	0.048	[-0.070 - 0.074]	0.000	1350	0.962	0.962
Left Postcentral	-0.053	0.014	-1.862	[-0.113 - 0.008]	0.000	1336	0.084	0.204
Left Posterior Cingulate	*-0.073*	*0.015*	*-2.489*	*[-0.136 - -0.010]*	*0.000*	*1363*	*0.026*	*0.117*
Left Precentral	*-0.083*	*0.015*	*-2.698*	*[-0.147 - -0.017]*	*0.000*	*1344*	*0.017*	*0.117*
Left Precuneus	-0.056	0.018	-1.505	[-0.135 - 0.024]	29.407	1337	0.154	0.309
Left Rostral Anterior Cingulate	0.016	0.017	0.480	[-0.055 - 0.087]	0.000	1347	0.639	0.689
Left Rostral Middle Frontal	***-0.080***	***0.011***	***-3.788***	***[-0.125 - -0.035]***	***0.000***	***1312***	***0.002***	***0.044***
Left Superior Frontal	***-0.101***	***0.010***	***-4.989***	***[-0.144 - -0.058]***	***0.000***	***1350***	***<0.001***	***0.014***
Left Superior Parietal	-0.023	0.017	-0.682	[-0.096 - 0.050]	8.394	1283	0.506	0.583
Left Superior Temporal	*-0.100*	*0.015*	*-3.300*	*[-0.164 - -0.035]*	*0.000*	*1197*	*0.005*	*0.072*
Left Supramarginal	*-0.073*	*0.015*	*-2.376*	*[-0.138 - -0.007]*	*0.000*	*1216*	*0.032*	*0.117*
Left Frontal Pole	-0.029	0.013	-1.098	[-0.086 - 0.028]	0.000	1368	0.291	0.388
Left Temporal Pole	-0.008	0.008	-0.480	[-0.043 - 0.027]	0.000	1329	0.639	0.689
Left Transverse Temporal	-0.060	0.014	-2.133	[-0.120 - 0.000]	0.000	1362	0.051	0.151
Left Insula	-0.038	0.018	-1.024	[-0.116 - 0.041]	11.502	1365	0.323	0.421
Right Bankssts	-0.043	0.021	-1.013	[-0.134 - 0.048]	28.028	1317	0.328	0.421

ROI	Partial *r*	SE	*t*-Score	95% CI	*I2*	*N*	*p-value*	FDR
Right Caudal Anterior Cingulate	0.010	0.019	0.268	[-0.072 - 0.092]	0.000	1361	0.793	0.842
Right Caudal Middle Frontal	***-0.099***	***0.013***	***-3.656***	***[-0.156 - -0.041]***	***0.000***	***1337***	***0.003***	***0.044***
Right Cuneus	-0.059	0.017	-1.723	[-0.132 - 0.015]	0.000	1316	0.107	0.242
Right Entorhinal	0.045	0.023	0.968	[-0.055 - 0.145]	52.099	1164	0.349	0.432
Right Fusiform	-0.034	0.015	-1.139	[-0.099 - 0.030]	0.000	1281	0.274	0.383
Right Inferior Parietal	-0.055	0.021	-1.291	[-0.146 - 0.037]	47.376	1226	0.218	0.366
Right Inferior Temporal	-0.042	0.015	-1.363	[-0.107 - 0.024]	0.000	1283	0.194	0.348
Right Isthmus Cingulate	-0.002	0.010	-0.077	[-0.045 - 0.042]	0.000	1351	0.940	0.954
Right Lateral Occipital	-0.052	0.014	-1.893	[-0.111 - 0.007]	0.000	1287	0.079	0.199
Right Lateral Orbitofrontal	-0.038	0.015	-1.310	[-0.100 - 0.024]	0.000	1333	0.211	0.366
Right Lingual	*-0.081*	*0.016*	*-2.581*	*[-0.147 - -0.014]*	*0.000*	*1350*	*0.022*	*0.117*
Right Medial Orbitofrontal	-0.029	0.015	-0.990	[-0.092 - 0.034]	0.000	1316	0.339	0.427
Right Middle Temporal	-0.029	0.017	-0.880	[-0.100 - 0.042]	0.000	1255	0.394	0.470
Right Parahippocampal	-0.024	0.018	-0.660	[-0.101 - 0.054]	0.000	1322	0.520	0.589
Right Paracentral	*-0.066*	*0.012*	*-2.794*	*[-0.116 - -0.015]*	*0.000*	*1361*	*0.014*	*0.117*
Right Pars Opercularis	*-0.079*	*0.015*	*-2.556*	*[-0.144 - -0.013]*	*0.000*	*1305*	*0.023*	*0.117*
Right Pars Orbitalis	-0.032	0.012	-1.282	[-0.085 - 0.021]	0.000	1336	0.221	0.366
Right Pars Triangularis	-0.056	0.014	-1.978	[-0.117 - 0.005]	0.000	1305	0.068	0.185
Right Pericalcarine	-0.062	0.017	-1.773	[-0.136 - 0.013]	0.000	1350	0.098	0.230
Right Postcentral	-0.040	0.013	-1.548	[-0.095 - 0.015]	0.000	1342	0.144	0.296
Right Posterior Cingulate	-0.034	0.012	-1.400	[-0.086 - 0.018]	0.000	1364	0.183	0.346
Right Precentral	*-0.065*	*0.012*	*-2.797*	*[-0.115 - -0.015]*	*0.000*	*1333*	*0.014*	*0.117*
Right Precuneus	-0.042	0.013	-1.633	[-0.097 - 0.013]	0.000	1333	0.125	0.274
Right Rostral Anterior Cingulate	-0.024	0.016	-0.757	[-0.094 - 0.045]	0.000	1327	0.462	0.541
Right Rostral Middle Frontal	-0.038	0.012	-1.551	[-0.091 - 0.015]	0.000	1302	0.143	0.296
Right Superior Frontal	***-0.090***	***0.010***	***-4.452***	***[-0.134 - -0.047]***	***0.000***	***1341***	***0.001***	***0.019***
Right Superior Parietal	-0.033	0.014	-1.223	[-0.091 - 0.025]	0.000	1314	0.242	0.370
Right Superior Temporal	*-0.075*	*0.015*	*-2.500*	*[-0.138 - -0.011]*	*0.000*	*1234*	*0.025*	*0.117*
Right Supramarginal	*-0.091*	*0.020*	*-2.248*	*[-0.176 - -0.004]*	*41.053*	*1223*	*0.041*	*0.134*
Right Frontal Pole	-0.029	0.013	-1.119	[-0.084 - 0.026]	0.000	1365	0.282	0.383
Right Temporal Pole	-0.020	0.016	-0.608	[-0.089 - 0.050]	0.000	1225	0.553	0.616
Right Transverse Temporal	-0.040	0.016	-1.214	[-0.109 - 0.030]	0.000	1363	0.245	0.370
Right Insula	*-0.066*	*0.015*	*-2.275*	*[-0.128 - -0.004]*	*0.000*	*1363*	*0.039*	*0.133*
Left Thickness	*-0.078*	*0.014*	*-2.820*	*[-0.136 - -0.019]*	*0.000*	*1370*	*0.014*	*1.000*
Right Thickness	*-0.084*	*0.012*	*-3.378*	*[-0.136 - -0.031]*	*0.000*	*1370*	*0.005*	*1.000*
Left Surface Area	-0.051	0.016	-1.620	[-0.118 - 0.017]	0.000	1370	0.128	1.000
Right Surface Area	-0.038	0.016	-1.201	[-0.106 - 0.030]	0.000	1370	0.249	1.000
Mean Bankssts	-0.056	0.019	-1.514	[-0.135 - 0.023]	0.000	1230	0.152	0.273
Mean Caudal Anterior Cingulate	0.029	0.015	0.967	[-0.035 - 0.092]	0.000	1354	0.350	0.426
Mean Caudal Middle Frontal	***-0.082***	***0.012***	***-3.317***	***[-0.135 - -0.029]***	***0.000***	***1323***	***0.005***	***0.043***
Mean Cuneus	-0.036	0.019	-0.965	[-0.116 - 0.044]	0.000	1243	0.351	0.426
Mean Entorhinal	0.023	0.025	0.477	[-0.082 - 0.128]	57.586	1137	0.641	0.681
Mean Fusiform	-0.063	0.016	-1.985	[-0.130 - 0.005]	0.000	1240	0.067	0.175
Mean Inferior Parietal	-0.054	0.021	-1.279	[-0.143 - 0.037]	45.036	1160	0.222	0.314
Mean Inferior Temporal	-0.030	0.013	-1.122	[-0.088 - 0.027]	0.000	1227	0.281	0.382
Mean Isthmus Cingulate	-0.017	0.010	-0.856	[-0.060 - 0.026]	0.000	1345	0.406	0.476
Mean Lateral Occipital	*-0.055*	*0.013*	*-2.157*	*[-0.109 - 0.000]*	*0.000*	*1237*	*0.049*	*0.138*
Mean Lateral Orbitofrontal	-0.041	0.015	-1.325	[-0.107 - 0.025]	0.000	1327	0.206	0.305
Mean Lingual	*-0.087*	*0.014*	*-3.064*	*[-0.147 - -0.026]*	*0.000*	*1328*	*0.008*	*0.057*
Mean Medial Orbitofrontal	-0.024	0.015	-0.789	[-0.088 - 0.041]	0.000	1298	0.443	0.502
Mean Middle Temporal	-0.045	0.017	-1.336	[-0.117 - 0.027]	0.000	1162	0.203	0.305

ROI	Partial *r*	SE	*t*-Score	95% CI	*I2*	*N*	*p-value*	FDR
Mean Parahippocampal	-0.050	0.019	-1.332	[-0.129 - 0.030]	38.397	1305	0.204	0.305
Mean Paracentral	*-0.063*	*0.013*	*-2.386*	*[-0.120 - -0.006]*	*0.000*	*1352*	*0.032*	*0.108*
Mean Pars Opercularis	*-0.095*	*0.017*	*-2.720*	*[-0.169 - -0.020]*	*28.507*	*1273*	*0.017*	*0.070*
Mean Pars Orbitalis	-0.041	0.011	-1.898	[-0.088 - 0.005]	0.000	1301	0.079	0.191
Mean Pars Triangularis	*-0.087*	*0.015*	*-2.911*	*[-0.150 - -0.023]*	*0.000*	*1256*	*0.011*	*0.065*
Mean Pericalcarine	-0.035	0.018	-0.987	[-0.111 - 0.041]	0.000	1340	0.341	0.426
Mean Postcentral	-0.048	0.014	-1.746	[-0.106 - 0.011]	0.000	1316	0.103	0.218
Mean Posterior Cingulate	*-0.058*	*0.013*	*-2.304*	*[-0.112 - -0.004]*	*0.000*	*1358*	*0.037*	*0.115*
Mean Precentral	***-0.088***	***0.013***	***-3.326***	***[-0.144 - -0.031]***	***0.000***	***1318***	***0.005***	***0.043***
Mean Precuneus	-0.047	0.016	-1.468	[-0.115 - 0.022]	2.046	1311	0.164	0.279
Mean Rostral Anterior Cingulate	-0.004	0.018	-0.111	[-0.081 - 0.073]	0.000	1309	0.913	0.913
Mean Rostral Middle Frontal	*-0.063*	*0.011*	*-2.833*	*[-0.110 - -0.015]*	*0.000*	*1263*	*0.013*	*0.065*
Mean Superior Frontal	***-0.098***	***0.010***	***-4.978***	***[-0.139 - -0.056]***	***0.000***	***1325***	***<0.001***	***0.007***
Mean Superior Parietal	-0.025	0.017	-0.756	[-0.097 - 0.046]	0.000	1248	0.462	0.507
Mean Superior Temporal	***-0.105***	***0.014***	***-3.780***	***[-0.164 - -0.046]***	***0.000***	***1132***	***0.002***	***0.034***
Mean Supramarginal	*-0.083*	*0.017*	*-2.442*	*[-0.155 - -0.010]*	*0.000*	*1147*	*0.028*	*0.107*
Mean Frontal Pole	-0.032	0.010	-1.622	[-0.075 - 0.010]	0.000	1363	0.127	0.245
Mean Temporal Pole	-0.007	0.013	-0.275	[-0.065 - 0.050]	0.000	1206	0.788	0.812
Mean Transverse Temporal	-0.055	0.015	-1.804	[-0.119 - 0.010]	0.000	1356	0.093	0.210
Mean Insula	-0.056	0.017	-1.610	[-0.130 - 0.019]	0.000	1358	0.130	0.245

Note: nominally significant results are italicized and FDR significant results are italicized and bolded

Supplementary Table S13 Associations between Subcortical Volumes and SANS Total

ROI	Partial *r*	SE	*t*-score	CI	*I2*	*N*	*p*-value	FDR
Left Lateral Ventricle	*0.050*	*0.009*	*2.683*	*[0.010 - 0.090]*	*0.000*	*1355*	*0.018*	*0.143*
Right Lateral Ventricle	*0.061*	*0.011*	*2.768*	*[0.014 - 0.109]*	*0.000*	*1355*	*0.015*	*0.143*
Left Thalamus	-0.022	0.016	-0.696	[-0.089 - 0.046]	0.000	1344	0.498	0.612
Right Thalamus	-0.047	0.020	-1.187	[-0.132 - 0.038]	12.938	1347	0.255	0.461
Left Caudate	0.033	0.014	1.128	[-0.029 - 0.094]	0.000	1345	0.278	0.461
Right Caudate	0.028	0.013	1.054	[-0.029 - 0.086]	0.000	1348	0.310	0.461
Left Putamen	0.012	0.014	0.410	[-0.049 - 0.072]	0.000	1347	0.688	0.787
Right Putamen	-0.006	0.014	-0.212	[-0.066 - 0.054]	0.000	1347	0.835	0.890
Left Pallidum	0.032	0.023	0.698	[-0.066 - 0.129]	74.360	1341	0.497	0.612
Right Pallidum	0.043	0.020	1.037	[-0.045 - 0.130]	54.829	1348	0.317	0.461
Left Hippocampus	-0.043	0.015	-1.450	[-0.107 - 0.021]	0.000	1339	0.169	0.451
Right Hippocampus	-0.048	0.013	-1.818	[-0.104 - 0.009]	0.000	1349	0.091	0.290
Left Amygdala	-0.003	0.015	-0.109	[-0.066 - 0.060]	0.000	1345	0.915	0.915
Right Amygdala	-0.051	0.022	-1.162	[-0.145 - 0.044]	41.363	1349	0.265	0.461
Left Accumbens	-0.072	0.018	-2.038	[-0.147 - 0.004]	0.000	1329	0.061	0.256
Right Accumbens	-0.059	0.015	-2.011	[-0.121 - 0.004]	0.000	1335	0.064	0.256
Mean Lateral Ventricle	*0.057*	*0.010*	*2.838*	*[0.014 - 0.100]*	*0.000*	*1355*	*0.013*	*0.093*

Mean Thalamus	-0.033	0.017	-0.943	[-0.107 - 0.042]	0.000	1342	0.362	0.413
Mean Caudate	0.031	0.013	1.159	[-0.026 - 0.088]	0.000	1343	0.266	0.413
Mean Putamen	0.005	0.014	0.180	[-0.055 - 0.065]	0.000	1341	0.860	0.860
Mean Pallidum	0.040	0.020	1.012	[-0.045 - 0.123]	0.000	1335	0.329	0.413
Mean Hippocampus	-0.043	0.014	-1.561	[-0.102 - 0.016]	0.000	1335	0.141	0.376
Mean Amygdala	-0.035	0.017	-1.014	[-0.110 - 0.039]	0.000	1341	0.328	0.413
Mean Accumbens	*-0.078*	*0.015*	*-2.545*	*[-0.143 - -0.012]*	*0.000*	*1318*	*0.023*	*0.093*

Note: nominally significant results are italicized and FDR significant results are italicized and bolded

Supplementary Table S14 Associations between Subcortical Volumes and SANS MAP

ROI	Partial *r*	SE	*t*-score	CI	*I*2	*N*	*p*-value	FDR
Left Lateral Ventricle	0.031	0.012	1.320	[-0.020 - 0.082]	0.000	1355	0.208	0.380
Right Lateral Ventricle	0.040	0.016	1.283	[-0.027 - 0.107]	0.000	1355	0.220	0.380
Left Thalamus	-0.007	0.012	-0.308	[-0.058 - 0.044]	0.000	1344	0.763	0.763
Right Thalamus	-0.038	0.016	-1.233	[-0.105 - 0.028]	0.000	1347	0.238	0.380
Left Caudate	0.029	0.014	1.052	[-0.030 - 0.088]	0.000	1345	0.311	0.452
Right Caudate	0.025	0.013	0.983	[-0.029 - 0.078]	0.000	1348	0.342	0.456
Left Putamen	0.022	0.014	0.787	[-0.037 - 0.081]	0.000	1347	0.445	0.547
Right Putamen	0.017	0.012	0.715	[-0.035 - 0.069]	0.000	1347	0.487	0.556
Left Pallidum	0.028	0.021	0.657	[-0.063 - 0.118]	68.092	1341	0.522	0.556
Right Pallidum	0.059	0.016	1.891	[-0.008 - 0.126]	0.000	1348	0.080	0.267
Left Hippocampus	-0.041	0.015	-1.405	[-0.103 - 0.022]	0.000	1339	0.182	0.380
Right Hippocampus	-0.039	0.015	-1.280	[-0.105 - 0.027]	0.000	1349	0.221	0.380
Left Amygdala	-0.048	0.013	-1.863	[-0.102 - 0.007]	0.000	1345	0.084	0.267
Right Amygdala	-0.075	0.019	-2.002	[-0.155 - 0.005]	15.320	1349	0.065	0.267
Left Accumbens	-0.065	0.016	-2.018	[-0.134 - 0.004]	0.000	1329	0.063	0.267
Right Accumbens	*-0.049*	*0.011*	*-2.246*	*[-0.096 - -0.002]*	*0.000*	*1335*	*0.041*	*0.267*
Mean Lateral Ventricle	0.037	0.014	1.346	[-0.022 - 0.096]	0.000	1355	0.200	0.434
Mean Thalamus	-0.021	0.014	-0.753	[-0.079 - 0.038]	0.000	1342	0.464	0.464
Mean Caudate	0.026	0.013	1.019	[-0.029 - 0.080]	0.000	1343	0.326	0.434
Mean Putamen	0.022	0.014	0.803	[-0.037 - 0.081]	0.000	1341	0.435	0.464
Mean Pallidum	0.039	0.019	1.026	[-0.042 - 0.119]	0.000	1335	0.322	0.434
Mean Hippocampus	-0.038	0.015	-1.289	[-0.102 - 0.025]	0.000	1335	0.218	0.434
Mean Amygdala	-0.069	0.016	-2.118	[-0.137 - 0.001]	0.000	1341	0.053	0.210
Mean Accumbens	*-0.066*	*0.013*	*-2.469*	*[-0.123 - -0.009]*	*0.000*	*1318*	*0.027*	*0.210*

Note: nominally significant results are italicized and FDR significant results are italicized and bolded

Supplementary Table S15 Associations between Subcortical Volumes and SANS Anhedonia (Factor 1)

ROI	Partial *r*	SE	*t*-score	CI	*I*2	*N*	*p*-value	FDR
Left Lateral Ventricle	0.010	0.012	0.424	[-0.041 - 0.061]	0.000	1355	0.678	0.933
Right Lateral Ventricle	0.026	0.015	0.860	[-0.038 - 0.089]	0.000	1355	0.404	0.905
Left Thalamus	0.004	0.016	0.121	[-0.063 - 0.070]	0.000	1344	0.905	0.933
Right Thalamus	-0.026	0.019	-0.677	[-0.108 - 0.056]	0.000	1347	0.509	0.905
Left Caudate	0.024	0.015	0.811	[-0.039 - 0.087]	0.000	1345	0.431	0.905
Right Caudate	0.021	0.014	0.751	[-0.040 - 0.083]	0.000	1348	0.465	0.905
Left Putamen	0.003	0.016	0.086	[-0.068 - 0.073]	0.000	1347	0.933	0.933
Right Putamen	-0.007	0.020	-0.167	[-0.094 - 0.081]	36.450	1347	0.870	0.933
Left Pallidum	0.005	0.018	0.152	[-0.070 - 0.081]	0.000	1341	0.881	0.933
Right Pallidum	0.043	0.015	1.453	[-0.020 - 0.106]	0.000	1348	0.168	0.905
Left Hippocampus	-0.013	0.017	-0.380	[-0.087 - 0.061]	0.000	1339	0.710	0.933
Right Hippocampus	-0.015	0.020	-0.386	[-0.100 - 0.069]	0.000	1349	0.705	0.933
Left Amygdala	-0.029	0.014	-1.020	[-0.090 - 0.032]	0.000	1345	0.325	0.905
Right Amygdala	-0.040	0.018	-1.122	[-0.117 - 0.037]	0.000	1349	0.281	0.905
Left Accumbens	-0.029	0.020	-0.727	[-0.115 - 0.057]	23.432	1329	0.479	0.905
Right Accumbens	-0.045	0.016	-1.427	[-0.113 - 0.023]	0.000	1335	0.176	0.905
Mean Lateral Ventricle	0.019	0.013	0.717	[-0.038 - 0.076]	0.000	1355	0.485	0.815
Mean Thalamus	-0.008	0.018	-0.233	[-0.083 - 0.067]	0.000	1342	0.819	0.936
Mean Caudate	0.024	0.014	0.841	[-0.037 - 0.085]	0.000	1343	0.415	0.815
Mean Putamen	0.000	0.018	0.010	[-0.076 - 0.077]	0.000	1341	0.992	0.992
Mean Pallidum	0.023	0.017	0.677	[-0.049 - 0.095]	0.000	1335	0.510	0.815
Mean Hippocampus	-0.014	0.018	-0.369	[-0.092 - 0.065]	0.000	1335	0.717	0.936
Mean Amygdala	-0.040	0.017	-1.163	[-0.113 - 0.034]	0.000	1341	0.264	0.815
Mean Accumbens	-0.046	0.018	-1.290	[-0.121 - 0.030]	1.414	1318	0.218	0.815

Note: nominally significant results are italicized and FDR significant results are italicized and bolded

Supplementary Table S16 Associations between Subcortical Volumes and SANS Asociality (Factor 2)

ROI	Partial *r*	SE	*t*-score	CI	*I*2	*N*	*p*-value	FDR
Left Lateral Ventricle	0.025	0.011	1.081	[-0.024 - 0.073]	0.000	1355	0.298	0.476
Right Lateral Ventricle	0.034	0.015	1.148	[-0.029 - 0.097]	0.000	1355	0.270	0.476
Left Thalamus	0.013	0.013	0.504	[-0.042 - 0.069]	0.000	1344	0.622	0.765
Right Thalamus	-0.011	0.015	-0.358	[-0.077 - 0.055]	0.000	1347	0.726	0.830
Left Caudate	0.036	0.016	1.145	[-0.032 - 0.103]	0.000	1345	0.271	0.476
Right Caudate	0.029	0.014	1.015	[-0.032 - 0.089]	0.000	1348	0.327	0.476
Left Putamen	0.054	0.016	1.715	[-0.014 - 0.121]	0.000	1347	0.108	0.355
Right Putamen	0.042	0.013	1.552	[-0.016 - 0.099]	0.000	1347	0.143	0.381
Left Pallidum	0.035	0.023	0.742	[-0.065 - 0.134]	0.000	1341	0.470	0.627
Right Pallidum	0.089	0.026	1.702	[-0.023 - 0.198]	78.521	1348	0.111	0.355
Left Hippocampus	-0.005	0.015	-0.156	[-0.068 - 0.058]	0.000	1339	0.878	0.937

Right Hippocampus	0.002	0.017	0.047	[-0.069 - 0.072]	0.000	1349	0.963	0.963
Left Amygdala	-0.029	0.013	-1.105	[-0.085 - 0.027]	0.000	1345	0.288	0.476
Right Amygdala	-0.064	0.016	-1.987	[-0.133 - 0.005]	0.000	1349	0.067	0.355
Left Accumbens	-0.071	0.017	-2.046	[-0.144 - 0.003]	0.000	1329	0.060	0.355
Right Accumbens	*-0.047*	*0.010*	*-2.375*	*[-0.090 - -0.005]*	*0.000*	*1335*	*0.032*	*0.355*
Mean Lateral Ventricle	0.030	0.013	1.164	[-0.025 - 0.086]	0.000	1355	0.264	0.423
Mean Thalamus	0.006	0.014	0.193	[-0.056 - 0.067]	0.000	1342	0.850	0.926
Mean Caudate	0.031	0.015	1.038	[-0.033 - 0.096]	0.000	1343	0.317	0.423
Mean Putamen	0.051	0.014	1.755	[-0.011 - 0.112]	0.000	1341	0.101	0.286
Mean Pallidum	0.056	0.026	1.055	[-0.058 - 0.168]	0.000	1335	0.309	0.423
Mean Hippocampus	0.003	0.016	0.095	[-0.067 - 0.073]	41.150	1335	0.926	0.926
Mean Amygdala	-0.051	0.015	-1.721	[-0.115 - 0.013]	0.000	1341	0.107	0.286
Mean Accumbens	*-0.065*	*0.014*	*-2.308*	*[-0.126 - -0.005]*	*0.000*	*1318*	*0.037*	*0.286*

Supplementary Table S17 Associations between Subcortical Volumes and SANS Avolition (Factor 3)

ROI	Partial *r*	SE	*t*-score	CI	*I*2	*N*	*p*-value	FDR
Left Lateral Ventricle	0.044	0.014	1.571	[-0.016 - 0.103]	0.000	1355	0.138	0.277
Right Lateral Ventricle	0.041	0.015	1.406	[-0.022 - 0.103]	0.000	1355	0.181	0.323
Left Thalamus	-0.039	0.011	-1.758	[-0.085 - 0.008]	0.000	1344	0.101	0.230
Right Thalamus	*-0.069*	*0.012*	*-2.776*	*[-0.122 - -0.016]*	*0.000*	*1347*	*0.015*	*0.059*
Left Caudate	0.008	0.012	0.331	[-0.044 - 0.060]	0.000	1345	0.746	0.796
Right Caudate	0.006	0.010	0.330	[-0.035 - 0.047]	0.000	1348	0.746	0.796
Left Putamen	-0.019	0.013	-0.764	[-0.073 - 0.035]	0.000	1347	0.457	0.563
Right Putamen	-0.015	0.008	-1.005	[-0.048 - 0.017]	0.000	1347	0.332	0.471
Left Pallidum	-0.006	0.011	-0.257	[-0.054 - 0.043]	0.000	1341	0.801	0.801
Right Pallidum	0.015	0.008	0.960	[-0.019 - 0.049]	0.000	1348	0.353	0.471
Left Hippocampus	***-0.092***	***0.011***	***-4.232***	***[-0.138 - -0.045]***	***0.000***	***1339***	***0.001***	***0.007***
Right Hippocampus	***-0.096***	***0.011***	***-4.317***	***[-0.142 - -0.048]***	***0.000***	***1349***	***0.001***	***0.007***
Left Amygdala	*-0.064*	*0.012*	*-2.784*	*[-0.114 - -0.015]*	*0.000*	*1345*	*0.015*	*0.059*
Right Amygdala	*-0.080*	*0.016*	*-2.437*	*[-0.150 - -0.010]*	*0.000*	*1349*	*0.029*	*0.092*
Left Accumbens	-0.054	0.014	-1.922	[-0.113 - 0.006]	0.000	1329	0.075	0.200
Right Accumbens	-0.032	0.015	-1.099	[-0.095 - 0.031]	0.000	1335	0.290	0.465
Mean Lateral Ventricle	0.044	0.014	1.541	[-0.017 - 0.104]	0.000	1355	0.146	0.233
Mean Thalamus	*-0.056*	*0.012*	*-2.401*	*[-0.105 - -0.006]*	*0.000*	*1342*	*0.031*	*0.082*
Mean Caudate	0.004	0.011	0.205	[-0.042 - 0.050]	0.000	1343	0.840	0.854
Mean Putamen	-0.015	0.011	-0.646	[-0.064 - 0.034]	0.000	1341	0.529	0.705
Mean Pallidum	0.004	0.011	0.187	[-0.041 - 0.049]	0.000	1335	0.854	0.854
Mean Hippocampus	***-0.096***	***0.011***	***-4.522***	***[-0.141 - -0.051]***	***0.000***	***1335***	***<0.001***	***0.004***
Mean Amygdala	*-0.079*	*0.014*	*-2.774*	*[-0.139 - -0.018]*	*0.000*	*1341*	*0.015*	*0.060*
Mean Accumbens	-0.054	0.014	-1.971	[-0.112 - 0.005]	0.000	1318	0.069	0.138

Note: nominally significant results are italicized and FDR significant results are italicized and bolded

Supplementary Table S18 Associations between Subcortical Volumes and SANS EXP

ROI	Partial *r*	SE	*t*-score	CI	*I*2	*N*	*p*-value	FDR
Left Lateral Ventricle	0.043	0.013	1.646	[-0.013 - 0.098]	0.000	1355	0.122	0.578
Right Lateral Ventricle	0.055	0.014	2.034	[-0.003 - 0.113]	0.000	1355	0.061	0.578
Left Thalamus	-0.023	0.017	-0.683	[-0.095 - 0.049]	0.000	1344	0.506	0.578
Right Thalamus	-0.047	0.023	-1.011	[-0.144 - 0.052]	53.318	1347	0.329	0.578
Left Caudate	0.027	0.014	0.946	[-0.034 - 0.088]	0.000	1345	0.360	0.578
Right Caudate	0.025	0.014	0.901	[-0.034 - 0.083]	0.000	1348	0.383	0.578
Left Putamen	-0.002	0.016	-0.072	[-0.071 - 0.067]	0.000	1347	0.943	0.943
Right Putamen	-0.030	0.016	-0.941	[-0.099 - 0.039]	0.000	1347	0.362	0.578
Left Pallidum	0.028	0.018	0.782	[-0.049 - 0.105]	0.000	1341	0.447	0.578
Right Pallidum	0.025	0.018	0.720	[-0.050 - 0.101]	0.000	1348	0.483	0.578
Left Hippocampus	-0.027	0.015	-0.864	[-0.092 - 0.039]	0.000	1339	0.402	0.578
Right Hippocampus	-0.033	0.013	-1.266	[-0.090 - 0.023]	0.000	1349	0.226	0.578
Left Amygdala	0.041	0.015	1.375	[-0.023 - 0.105]	0.000	1345	0.191	0.578
Right Amygdala	-0.016	0.022	-0.367	[-0.108 - 0.077]	46.148	1349	0.719	0.767
Left Accumbens	-0.062	0.016	-1.884	[-0.131 - 0.009]	0.000	1329	0.081	0.578
Right Accumbens	-0.045	0.016	-1.431	[-0.112 - 0.022]	16.963	1335	0.174	0.578
Mean Lateral Ventricle	0.050	0.013	1.859	[-0.008 - 0.107]	0.000	1355	0.084	0.336
Mean Thalamus	-0.030	0.019	-0.792	[-0.110 - 0.051]	0.000	1342	0.442	0.589
Mean Caudate	0.029	0.014	1.052	[-0.030 - 0.087]	0.000	1343	0.311	0.589
Mean Putamen	-0.015	0.016	-0.454	[-0.083 - 0.054]	0.000	1341	0.657	0.750
Mean Pallidum	0.031	0.018	0.861	[-0.047 - 0.109]	0.000	1335	0.403	0.589
Mean Hippocampus	-0.027	0.014	-0.979	[-0.087 - 0.032]	0.000	1335	0.344	0.589
Mean Amygdala	0.007	0.017	0.217	[-0.065 - 0.079]	0.000	1341	0.831	0.831
Mean Accumbens	*-0.068*	*0.015*	*-2.253*	*[-0.133 - -0.003]*	*0.000*	*1318*	*0.041*	*0.327*

Note: nominally significant results are italicized and FDR significant results are italicized and bolded

Supplementary Table S19 Associations between Subcortical Volumes and SANS Blunted Affect (Factor 4)

ROI	Partial *r*	SE	*t*-score	CI	*I*2	*N*	*p*-value	FDR
Left Lateral Ventricle	0.041	0.014	1.527	[-0.017 - 0.099]	0.000	1355	0.149	0.597
Right Lateral Ventricle	0.053	0.014	1.920	[-0.006 - 0.112]	0.000	1355	0.076	0.597
Left Thalamus	-0.010	0.018	-0.296	[-0.086 - 0.065]	0.000	1344	0.772	0.882
Right Thalamus	-0.040	0.025	-0.803	[-0.145 - 0.066]	66.436	1347	0.435	0.674
Left Caudate	0.031	0.014	1.125	[-0.028 - 0.090]	0.000	1345	0.279	0.674
Right Caudate	0.029	0.014	1.025	[-0.031 - 0.088]	0.000	1348	0.323	0.674
Left Putamen	0.004	0.017	0.110	[-0.070 - 0.077]	0.000	1347	0.914	0.914
Right Putamen	-0.026	0.017	-0.773	[-0.099 - 0.046]	0.000	1347	0.452	0.674

Left Pallidum	0.029	0.020	0.722	[-0.057 - 0.115]	0.000	1341	0.482	0.674
Right Pallidum	0.027	0.019	0.716	[-0.053 - 0.106]	0.000	1348	0.486	0.674
Left Hippocampus	-0.013	0.015	-0.433	[-0.080 - 0.053]	0.000	1339	0.672	0.827
Right Hippocampus	-0.019	0.014	-0.684	[-0.080 - 0.042]	0.000	1349	0.505	0.674
Left Amygdala	0.053	0.016	1.695	[-0.014 - 0.119]	0.000	1345	0.112	0.597
Right Amygdala	-0.008	0.023	-0.172	[-0.107 - 0.091]	59.701	1349	0.866	0.914
Left Accumbens	-0.065	0.017	-1.935	[-0.137 - 0.007]	0.000	1329	0.073	0.597
Right Accumbens	-0.037	0.016	-1.146	[-0.107 - 0.033]	28.732	1335	0.271	0.674
Mean Lateral Ventricle	0.048	0.014	1.741	[-0.011 - 0.107]	0.000	1355	0.104	0.414
Mean Thalamus	-0.018	0.020	-0.443	[-0.103 - 0.068]	8.355	1342	0.664	0.712
Mean Caudate	0.033	0.014	1.219	[-0.025 - 0.091]	0.000	1343	0.243	0.648
Mean Putamen	-0.014	0.018	-0.377	[-0.092 - 0.064]	18.043	1341	0.712	0.712
Mean Pallidum	0.033	0.020	0.824	[-0.052 - 0.117]	0.000	1335	0.424	0.712
Mean Hippocampus	-0.013	0.014	-0.464	[-0.075 - 0.048]	0.000	1335	0.650	0.712
Mean Amygdala	0.023	0.019	0.611	[-0.058 - 0.104]	28.867	1341	0.551	0.712
Mean Accumbens	-0.064	0.016	-1.989	[-0.132 - 0.005]	11.193	1318	0.067	0.414

Note: nominally significant results are italicized and FDR significant results are italicized and bolded

Supplementary Table S20 Associations between Subcortical Volumes and SANS Alogia

ROI	Partial *r*	SE	*t*-score	CI	*I*2	*N*	*p*-value	FDR
Left Lateral Ventricle	0.034	0.012	1.474	[-0.016 - 0.084]	0.000	1355	0.163	0.325
Right Lateral Ventricle	0.045	0.013	1.740	[-0.010 - 0.100]	0.000	1355	0.104	0.237
Left Thalamus	-0.054	0.015	-1.813	[-0.118 - 0.010]	0.000	1344	0.091	0.237
Right Thalamus	-0.064	0.016	-2.059	[-0.131 - 0.003]	0.000	1347	0.059	0.237
Left Caudate	0.003	0.015	0.112	[-0.060 - 0.067]	0.000	1345	0.913	0.962
Right Caudate	0.001	0.013	0.048	[-0.053 - 0.055]	0.000	1348	0.962	0.962
Left Putamen	-0.022	0.015	-0.736	[-0.087 - 0.043]	0.000	1347	0.474	0.689
Right Putamen	-0.033	0.015	-1.127	[-0.097 - 0.030]	0.000	1347	0.279	0.446
Left Pallidum	0.013	0.015	0.424	[-0.051 - 0.077]	0.000	1341	0.678	0.784
Right Pallidum	0.016	0.015	0.541	[-0.047 - 0.078]	0.000	1348	0.597	0.784
Left Hippocampus	-0.060	0.016	-1.876	[-0.128 - 0.009]	0.000	1339	0.082	0.237
Right Hippocampus	*-0.066*	*0.012*	*-2.778*	*[-0.116 - -0.015]*	*0.000*	*1349*	*0.015*	*0.237*
Left Amygdala	-0.009	0.011	-0.413	[-0.059 - 0.040]	0.000	1345	0.686	0.784
Right Amygdala	-0.054	0.015	-1.836	[-0.116 - 0.009]	0.000	1349	0.088	0.237
Left Accumbens	-0.028	0.012	-1.154	[-0.081 - 0.024]	0.000	1329	0.268	0.446
Right Accumbens	-0.052	0.014	-1.874	[-0.111 - 0.008]	0.000	1335	0.082	0.237
Mean Lateral Ventricle	0.040	0.012	1.612	[-0.013 - 0.092]	0.000	1355	0.129	0.258
Mean Thalamus	-0.059	0.014	-2.047	[-0.120 - 0.003]	0.000	1342	0.060	0.167
Mean Caudate	0.003	0.013	0.123	[-0.054 - 0.061]	0.000	1343	0.904	0.904
Mean Putamen	-0.029	0.015	-0.974	[-0.092 - 0.034]	0.000	1341	0.347	0.462

Mean Pallidum	0.016	0.015	0.529	[-0.048 - 0.079]	0.000	1335	0.605	0.692
Mean Hippocampus	-0.062	0.014	-2.126	[-0.124 - 0.001]	0.000	1335	0.052	0.167
Mean Amygdala	-0.034	0.013	-1.342	[-0.088 - 0.020]	0.000	1341	0.201	0.322
Mean Accumbens	-0.050	0.012	-2.022	[-0.103 - 0.003]	0.000	1318	0.063	0.167

Note: nominally significant results are italicized and FDR significant results are italicized and bolded

Supplementary Table S21. Associations Between Symptom Correlation Effect Size Maps and Receptor Distribution Maps

	**SANS Total**		**MAP**		**Anhedonia**		**Asociality**		**Avolition**		**EXP**		**Blunted Affect**		**Alogia**	
	Corr	*p*	Corr	*p*	Corr	*p*	Corr	*p*	Corr	*p*	Corr	*p*	Corr	*p*	Corr	*p*
5HT1a	0.047	0.319	0.002	0.469	-0.149	0.120	0.182	0.085	-0.144	0.149	0.127	0.139	0.184	0.053	0.056	0.332
5HT1b	-0.183	0.099	-0.219	0.070	-0.097	0.242	_-0.273*_	0.021	-0.159	0.139	-0.116	0.221	-0.131	0.211	-0.152	0.122
5HT2a	-0.114	0.149	_-0.222*_	0.028	-0.176	0.066	_-0.300*_	0.005	-0.103	0.162	-0.081	0.204	-0.057	0.244	-0.158	0.104
5HT4	-0.062	0.225	-0.109	0.174	-0.121	0.143	-0.041	0.340	-0.138	0.105	-0.001	0.413	0.065	0.384	-0.053	0.314
5HT6	-0.086	0.200	-0.156	0.108	-0.101	0.201	-0.155	0.116	-0.145	0.114	-0.006	0.429	0.009	0.560	-0.140	0.143
5HTT	_0.271*_	0.018	_0.311*_	0.002	0.215	0.050	_0.432*_	0.000	0.120	0.174	_0.228*_	0.044	_0.227_	0.040	0.185	0.065
A4B2	_-0.329*_	0.008	_-0.339*_	0.009	_-0.363*_	0.006	_-0.285*_	0.019	_-0.313*_	0.012	_-0.249*_	0.042	_-0.252*_	0.037	-0.169	0.101
CB1	_-0.302*_	0.008	_-0.357*_	0.004	_-0.363*_	0.001	-0.212	0.050	_-0.377*_	0.001	-0.138	0.106	-0.090	0.169	-0.117	0.171
D1	0.124	0.098	0.081	0.226	0.064	0.261	0.189	0.053	-0.045	0.475	0.153	0.062	_0.177*_	0.038	0.017	0.450
D2	-0.063	0.318	-0.110	0.192	-0.163	0.107	0.041	0.371	-0.210	0.053	0.074	0.254	0.134	0.129	0.016	0.461
DAT	_0.233*_	0.034	_0.206*_	0.044	0.175	0.063	_0.299*_	0.007	0.042	0.311	_0.275*_	0.013	_0.302*_	0.007	0.166	0.103
GABAa	0.124	0.187	0.115	0.205	_0.228*_	0.041	0.053	0.358	0.106	0.204	0.083	0.260	0.082	0.291	-0.066	0.300
H3	_-0.259*_	0.033	-0.261	0.056	-0.240	0.050	-0.162	0.134	_-0.263*_	0.041	-0.184	0.093	-0.162	0.129	-0.218	0.057
M1	-0.212	0.085	_-0.255*_	0.031	-0.208	0.066	-0.212	0.055	-0.208	0.078	-0.187	0.106	-0.197	0.101	-0.197	0.064
mGluR5	-0.075	0.331	-0.051	0.417	-0.023	0.451	0.008	0.477	-0.109	0.249	-0.064	0.411	-0.039	0.480	-0.124	0.184
MOR	_-0.231*_	0.031	_-0.291*_	0.018	_-0.331*_	0.005	-0.141	0.140	_-0.350*_	0.004	-0.088	0.254	-0.048	0.371	-0.041	0.376
NET	-0.162	0.141	-0.093	0.267	-0.048	0.393	-0.065	0.335	-0.119	0.222	-0.163	0.135	-0.184	0.097	-0.122	0.169
NMDA	0.159	0.081	0.086	0.238	0.094	0.207	0.141	0.127	-0.004	0.550	_0.202*_	0.036	_0.208*_	0.029	0.085	0.253
VAChT	0.007	0.442	0.073	0.306	0.065	0.305	0.170	0.116	-0.056	0.368	0.011	0.427	0.023	0.368	-0.014	0.467

Note: Significant correlations denoted with * and italicized

Factor Score Formulas

SANS factor calculation. Factor computation was based on confirmatory factor analyses (Strauss & Ahmed, personal communication, January 6, 2020).

MAP = [Recreational Interests and Activities (score)*0.810] + [Sexual Activity (score)*0.641] + [Ability to Feel Intimacy and Closeness (score)*0.688] + [Relationships with Friends and Peers (score)*0.804] + [Grooming and Hygiene (score)*0.314] + [Inpersistence at Work or School (score)*0.553] + [Physical Anergia (score)*0.648]

Anhedonia Factor = Recreational Interests and Activities (score)*0.707

Asociality Factor = [Sexual Activity (score)*0.685] + [Ability to Feel Intimacy and Closeness (score)*0.736] + [Relationships with Friends and Peers (score)*0.868]

Avolition Factor = [Grooming and Hygiene (score)*0.395] + [Inpersistence at Work or School (score)*0.666] + [Physical Anergia (score)*0.807]

EXP = [Unchanging Facial Expression (score)*0.831] + [Decreased Spontaneous Movements (score)*0.835] + [Paucity of Expressive Gestures (score)*0.901] + [Poor Eye Contact (score)*0.535] + [Affective Nonresponsivity (score)*0.654] + [Lack of Vocal Inflections (score)*0.765] + [Poverty of Speech (score)*0.775] + [Blocking (score)*0.291] + [Increased Latency of Response (score)*0.415]

Blunted Affect Factor = [Unchanging Facial Expression*0.835] + [Decreased Spontaneous Movements (score)*0.840] + [Paucity of Expressive Gestures (score)*0.904] + [Poor Eye Contact (score)*0.541] + [Affective Nonresponsivity (score)*0.658] + [Lack of Vocal Inflections (score)*0.770]

Alogia Factor = [Poverty of Speech (score)*0.867] + [Blocking (score)*0.313] + [Increased Latency of Response (score)*0.440]

Note. * denotes multiplication.
